# The effects of antibiotic use on the dynamics of the microbiome and resistome in pigs

**DOI:** 10.1186/s42523-023-00258-4

**Published:** 2023-08-21

**Authors:** Katrine Wegener Tams, Inge Larsen, Julie Elvekjær Hansen, Henrik Spiegelhauer, Alexander Damm Strøm-Hansen, Sophia Rasmussen, Anna Cäcilia Ingham, Lajos Kalmar, Iain Robert Louis Kean, Øystein Angen, Mark A. Holmes, Karl Pedersen, Lars Jelsbak, Anders Folkesson, Anders Rhod Larsen, Mikael Lenz Strube

**Affiliations:** 1 Department of Biotechnology and Biomedicine, Technical University of Denmark, 2800 Kgs. Lyngby, Denmark; 2 Department of Veterinary and Animal Sciences, University of Copenhagen, 1871 Copenhagen, Denmark; 3 Department of Veterinary Medicine, University of Cambridge, Cambridge, UK; 4 Department of Bacteria, Parasites and Fungi, Statens Serum Institut (SSI), 2300 Copenhagen, Denmark; 5 Department of Animal Health and Antimicrobial Strategies, National Veterinary Institute, 751 89 Uppsala, Sweden

**Keywords:** Antibiotic resistance, Pig farming, Microbiome, Metagenomes

## Abstract

Antibiotics are widely used in pig farming across the world which has led to concerns about the potential impact on human health through the selection of antibiotic resistant pathogenic bacteria. This worry has resulted in the development of a production scheme known as pigs Raised Without Antibiotics (RWA), in which pigs are produced in commercial farms, but are ear-tagged as RWA until slaughter unless they receive treatment, thus allowing the farmer to sell the pigs either as premium priced RWA or as conventional meat. Development of antibiotic resistance in pig farming has been studied in national surveys of antibiotic usage and resistance, as well as in experimental studies of groups of pigs, but not in individual pigs followed longitudinally in a commercial pig farm. In this study, a cohort of RWA designated pigs were sampled at 10 time points from birth until slaughter along with pen-mates treated with antibiotics at the same farm. From these samples, the microbiome, determined using 16S sequencing, and the resistome, as determined using qPCR for 82 resistance genes, was investigated, allowing us to examine the difference between RWA pigs and antibiotic treated pigs. We furthermore included 176 additional pigs from six different RWA farms which were sampled at the slaughterhouse as an endpoint to substantiate the cohort as well as for evaluation of intra-farm variability. The results showed a clear effect of age in both the microbiome and resistome composition from early life up until slaughter. As a function of antibiotic treatment, however, we observed a small but significant divergence between treated and untreated animals in their microbiome composition immediately following treatment, which disappeared before 8 weeks of age. The effect on the resistome was evident and an effect of treatment could still be detected at week 8. In animals sampled at the slaughterhouse, we observed no difference in the microbiome or the resistome as a result of treatment status but did see a strong effect of farm origin. Network analysis of co-occurrence of microbiome and resistome data suggested that some resistance genes may be transferred through mobile genetic elements, so we used Hi-C metagenomics on a subset of samples to investigate this. We conclude that antibiotic treatment has a differential effect on the microbiome vs. the resistome and that although resistance gene load is increased by antibiotic treatment load, this effect disappears before slaughter. More studies are needed to elucidate the optimal way to rear pigs without antibiotics.

**Supplementary Information:**

The online version contains supplementary material available at 10.1186/s42523-023-00258-4.

## Introduction

In 2020, 76 tonnes of antibiotics were used in the Danish pig farms to raise approximately 30 million pigs over the year, which makes pig farming the biggest sector using antibiotics in Danish society. In comparison, approximately 45 tonnes of antibiotics were used for human treatment in the same year in Denmark [12]. Antibiotics are mostly used in weaning piglets to treat post-weaning diarrhoea.

Apart from the clinical and societal effects of potential increases in resistant bacteria, the high use of antibiotics is a concern for consumers and in 2016, an initiative to Raise pigs Without Antibiotics (RWA) was adopted by the meat producer Danish Crown. On such farms, pigs are initially marked as RWA, but over the course of their life-time many (typically 30–40%) will individually receive antibiotics to treat infections and thus lose their “raised without antibiotics” status. These pigs, regardless of antibiotic treatment, have the same genetic background and are exposed to the same living conditions. Hence, RWA farms provides a unique opportunity to examine effects of antibiotic treatment in pigs.

Antibiotic resistant bacteria and auxiliary metabolic genes have been found to spread from farms to the broader environment, posing a potential zoonotic threat to human treatment opportunities [24, 28, 53]. These can be transferred directly from animal farms or through food to people and may subsequently be transferred to the bacteria in the human microbiome, serving as a potential source of more clinically relevant antimicrobial resistant bacteria [45].

The resistome is defined as the collection of all the antibiotic resistance genes of a given sample or environment [50] and can be considered as a feature of the microbiome. The presence of antibiotics in an environment will select positively for resistant bacteria as well as individual resistance genes and the constructs that carry them. Indeed, studies have observed that the level of resistance genes, e.g. the resistome, is increased in animals treated with antibiotics [22, 37], and also that early antibiotic treatment can have lasting effects on the resistome in weaned pigs [17]. The most commonly observed resistance genes in European pigs, including Denmark, are genes encoding resistance towards tetracycline, macrolides, β-lactams and aminoglycosides [37]. In order to quantify these genes, metagenomics analysis [30, 37] and high-throughput qPCR have been used [7, 11, 19]. Previous studies have also found a fundamental difference between individual farms, which may overshadow the effect of treatment [19].

In Danish pig farms, the general guidelines are to use first choice antibiotics (e.g., penicillins and sulfonamides). If these are ineffective, second choice antibiotics can be used (e.g., apramycin, gentamicin, and tetracyclines). The third choice antibiotics are strongly restricted for use in pigs as they are clinically critical for treatment in human diseases (fluoroquinolones, colistin, and 3rd and 4th generation cephalosporins) [35]. Although zinc has routinely and effectively been added to animal feed in many countries to combat intestinal infections, zinc oxide in feed has been banned from June 2022 by the European Commission, i.e. after this study was conducted [6]. A major concern was the accumulation of zinc as a heavy metal pollution on farm land due to spreading of pig manure and slurry, but the zinc may also have contributed to increased levels of antibiotic resistance and multiresistant bacteria [10, 36]. The RWA pigs in this study received zinc oxide the first 14 days after weaning as was customary at the time.

Pig microbiomes are affected by many factors, including age, host genes, breed, gender and castration of male pigs [51]. The microbiome becomes more diverse with age of the pig [46] and matures over time, but the most profound change in the pig microbiome is at weaning when the pigs are transitioning from sow milk to solid feed where e.g. the *Lactobacillaceae* family is drastically reduced in abundance [13]. A highly diverse microbiome contributes to the health of the pigs as it helps to exclude pathogens [16]. The microbiome becomes less diverse after antibiotic treatment and treatment at an early age can cause lasting changes of composition of the microbiomes in pigs [43].

As described above, antibiotics have been observed to cause perturbation of the microbiome, especially in young animals. The scale and temporal dynamics of microbiome and resistome have not been studied in detail, and hence, the aim of our study was to investigate to what degree antibiotic treatment causes changes in the microbiome and the resistome of pigs and if these changes remain over time. Moreover, we investigated if the temporal dynamics of the resistome and the microbiome was decoupled and, if so, which resistance genes were involved.

## Methods

### Study design

**Fig. 1:**
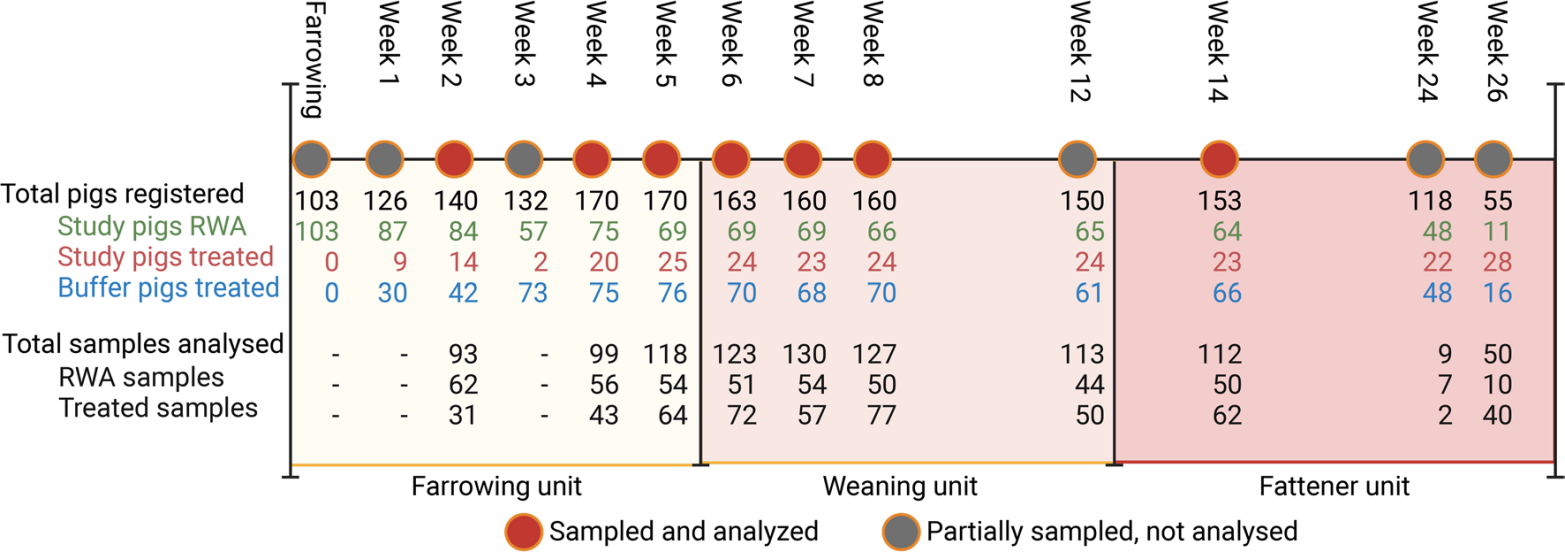
Sampling scheme of the study. From a cohort of 513 pigs, 103 initial RWA pigs were followed in detail along with 99 progressively enrolled pigs from an initial pool of 410 untreated buffer pigs. One hundred and three pigs were initially followed regardless of treatment (study pigs). For example, in week 2, 84 animals remained RWA, while 14 animals had received antibiotic treatment. In the buffer group, 42 animals had received antibiotic treatment by week 2 and where hence enrolled for sampling downstream, resulting in 140 total pigs registered in that week. Of these 140 pigs, 93 were successfully used for analyses, i.e. had full metadata and complete 16S and qPCR data. Due to logistics and internal movement of the pigs between farm units, some sampling weeks where either under-sampled (week 3) or disturbed by ongoing transfer of pigs between units (weeks 12, 24 and 26) and were hence excluded from statistical testing

The study was designed as a cohort study following a batch of pigs (e.g., born within the same week) from birth until 26 weeks of age and was carried out in a fully operational RWA farm with no intervention in the ongoing management routines. The farm had a fully integrated production system ensuring no mixing with pigs from other farms. As standard practice, all pigs were ear tagged with a RWA tag (Additional file 1: Figure S1) shortly after birth which was removed if pigs were treated with antibiotics at any time during the rearing. To ensure a reasonably random and representative selection of the population, every fifth pig (id no = 5, 10, 15, etc.) (n = 103) was clinically assessed from the day of birth and subsequently sampled at 2 weeks of age and at 4, 5, 6, 7, 8, 12, 14, 24 and 26 weeks of age (Fig. 1). As precautionary measure, the remaining pigs (n = 410) served as buffer group. Pigs from this buffer group were enrolled in the study, sampled and clinically assessed if they were treated with antibiotics in order to ensure more antibiotic-treated pigs in the case–control study. Ninety-nine pigs from the buffer group were enrolled during the sampling period after registration of missing RWA ear tags.

In order to increase data available for analysis in the final stage of the study, to provide data to assess the external validity of the study, and to conclude on the final effect of RWA-status, we sampled an additional 176 pigs from 6 different RWA farms at the slaughterhouse.

### Data collection at pig herd

All live-born pigs (n = 513) from a two-day period (12–13 April 2018) from 29 sows were included in the study and tagged with a unique identifier in addition to the RWA tag (Fig. 1). Additional details of the farm and pigs used in this study has been published by Lynegaard et al. [32] in which it is named ‘herd A’.

Of the 513 pigs born at this particular week (whole batch), 134 were treated with antibiotics at least once. The overall mortality was 11% at week 12 [32]. All of the animals of the batch were tagged as RWA, but only 103 of these were randomly selected and followed regardless of treatment status to represent the farm treatment patterns. The remaining 410 pigs (buffer group) were only enrolled for sampling if treated with antibiotics.

All treatments were given according to the farm’s standard practice and not influenced by the researchers. The treatment options included tetracyclines, macrolides, β-lactams or lincosamides for suckling piglets, and tetracyclines, macrolides, β-lactams or aminoglycosides for weaning pigs (Additional file 1: Table S1). The specific antibiotic treatment of each pig was only registered in weeks 2 and 3, but the overall antimicrobial consumption for sows and piglets (farrowing unit) in this farm was 1.3 animal daily doses (ADD) per 100 animals per day compared to 2.5 ADD for the average Danish farrowing unit. Prescriptions here were mainly due to reproductive, gastrointestinal and respiratory infections. The antimicrobial consumption for weaners in the farm was 1 ADD compared to 9 ADD for the average Danish weaner pig herd. The antimicrobial prescription for this age group were due to enteric diseases (https://vetstat.fvst.dk/vetstat/).

Since much of the sampling was performed on young animals, samples were collected as rectal swabs using sterile Dryswab® Fine Tip MW113 swabs (Medical Wire Equipment, Corsham, UK). rather than faecal samples. These were transferred to 3.6 mL Nunc® CryoTubes^®^ containing 1.5 mL phosphate-buffered saline (PBS) and stored under refrigeration until processing. The tubes were vortexed vigorously and the liquid contents stored at − 80 °C before further processing within 24 h after sampling.

Since RWA status of a given pig was subject to change at each sampling, RWA status was recorded at each sampling. At this farm, antibiotic treatment was mainly administered in weeks 2 and 3 and cross-referenced by treatment type. Due to logistic issues, no registration of subsequent treatment of these pigs was recorded although it likely occurred for several pigs, which may have somewhat added noise to the data. As is customary in RWA farms, pigs were not separated from untreated pigs when undergoing antibiotic treatment. All antibiotics were administered by injection and no antibiotics in the feed or water were administered at any time. It has previously been confirmed that injected antibiotics reach the intestinal lumen and leads to perturbation of the gut microbiome [41].

The pigs of the batch were born over two days and were moved from the farrowing unit to the weaning unit at ~ 4 weeks old as is customary in Danish pig stables. In the weaning unit, animals were housed in pens of 30–40 pigs in a stable of a total of 18 pens. Later, animals were moved to the slaughter unit on two separate days (~ 9 or ~ 12 weeks old) and the majority at ~ 12 weeks old and housed here at ~ 20 pigs per pen in a stable with 16 total pens. Representative data were recorded in weeks 2, 4, 5, 6, 7, 8 and 14 for comparative statistical analysis. Due to logistics and internal movement of the pigs between farm units, some sampling weeks where either under-sampled (week 3) or disturbed by ongoing transfer of pigs between units (weeks 12, 24 and 26) and were hence excluded from statistical testing.

### Data collection at slaughterhouse

Faecal samples (n = 176) were collected immediately after removal of plucks and intestines, while the carcass and earmark were still directly above for identification at the slaughterhouse line. Around 30 pigs from each farm consisting of 15 RWA pigs and 15 non-RWA pigs. Approximately 10–20 ml of faecal content was obtained from the rectum and stored at − 80 °C within 12 h.

### DNA extraction

Farm samples were extracted as described by Strube et al. [48]. Briefly, DNA was extracted on a Maxwell^®^16 Research Instrument System (Promega Corporation, Wisconsin, USA) according to the manufacturer’s instructions. Negative controls for each run were obtained by not adding any faecal sample material to one of the Eppendorf tubes. DNA from the slaughterhouse samples was extracted from ~ 250 mg faeces using DNeasy PowerSoil Pro Kit from Qiagen according to manufacturer’s guidelines (Qiagen, Hilden, Germany). Since the two sets of samples were extracted differently, direct comparisons between them were avoided.

### High-capacity quantitative PCR arrays

DNA concentration was measured on NanoDrop ND-1000 Spectrophotometer (NanoDrop Technologies, Thermo Fisher, Wilmington, DE, USA), diluted to 10 ng/μl in nuclease-free water (Qiagen) and stored at − 20 °C until further processing.

qPCR on 82 antibiotic resistance genes (ARGs) was performed with the Fluidigm HD Biomark system (Fluidigm Corporation, South San Francisco, CA, USA) using Taq Man Universal Mastermix (Applied Biosystem, Thermo Fischer Scientific) and EvaGreen (20X, Biotium, USA). The PCR program was: Thermal mix, at 50 °C for 2 min, 70 °C for 30 min, 25 °C for 10 min; UNG and Hot Start 50 °C for 2 min, 95 °C for 10 min; 35 cycles of 95 °C 15 s, 60 °C for one min; lastly a melting phase 60 °C for 30 s followed by raising the temp to 95 °C by 1 °C per 3 s. The primers were chosen as the most agriculturally abundant and/or clinically relevant ones described in the literature [25, 54]. All primers are listed in Additional file 1: Table S2. As positive controls, a pool of artificial DNA amplicons for all primer sets was included. Additionally, a reference control for plate-to-plate comparisons was included, containing a pool of extracted DNA from 33 different bacterial strains in equivalent volume and DNA concentration, along with 2 standardized faecal samples. Negative controls consisted of TE buffer and empty wells. 16S rRNA gene primers were included in triplicate as an internal reference, allowing us to normalize each gene to bacterial abundance. Samples with one or more 16S replicates with no signal were excluded (n = 8) from the analysis as well as triplicates with a coefficient of variation over 5 (n = 3). Very small (< 0.001) normalized gene values were set to 0.

The primers covered most clinically relevant antibiotic resistance genes, as detailed in Additional file 1: Table S2, together with one plasmid replication initiator protein and three IS elements associated with plasmids carrying antimicrobial resistance genes.

### 16S rRNA sequencing metataxonomics

Sequencing of the V3V4-region of the 16S rRNA gene was done on a Illumina MiSeq (Illumina Inc., San Diego, CA, USA) as previously described [23] and processing was performed using DADA2 [8], using the Silva reference database and species-level training set (v. 138) formatted for DADA2 [34]. The total number of samples was 1,093 (controls and reruns excluded), the mean input read count was 39,007, mean filtered and trimmed read count 29,695, read count after merge in DADA2 was 29,695, and the non-chimeric read count was 24,108. Samples with less than 1000 reads were re-sequenced and excluded if still below 1000 reads (n = 93).

### Hi-C metagenomics

In order to achieve broad coverage of the metagenome of the farm, w selected a seven pigs with different treatment statuses (none, early and late) at four time points, along with their corresponding 7 birth sows at 2 time points. These samples were then subjected to Hi-C metagenomics was performed. The HAM-ART Hi-C method ensures physical links between DNA molecules in close proximity, linking genetic regions to one another [26]. In the case of microbiome metagenomics, this will link plasmids to genomes, hence increasing the chance of connecting ARGs residing on plasmids to the chromosomes of specific bacteria. Briefly, samples were subjected to 2.5% formaldehyde to facilitate DNA crosslinking, after which they were quenched with glycine and sequenced and bioinformatically processed as described in [26].

### Statistics

Samples were removed from analysis only if having incomplete meta-data (n = 253). All statistics were carried out in R v4.1.1 using the vegan package [38].

Since pigs were treated with antibiotics at different time-points, each sampling week created an additional group, creating challenges in data analysis. Because of this, two different modelling approaches were then considered: (1) A simple model, where pigs belong to either the treated or untreated RWA group regardless of when they were treated or (2) time-of-treatment model, where pigs belong to an untreated RWA group or a time-of-treatment group according to the week they received antibiotics (week 1, 2, 3, 4, 5). In the time-of-treatment model, later treatment weeks were not included due to low group numbers (< 5 pigs per group). This model was only used to describe univariate analysis of ARGs (see below).

For multivariate analysis, only the simple model (treated vs untreated across time) was used due to underpowering of the time-of-treatment model. Using the time-of-treatment model did not result in different conclusions but was avoided due to underpowering of the groups and the complexities in interpreting a non-linear and multivariate model with increasing group numbers across time.

16S rRNA gene data (at the amplicon sequence variant (ASV) level) and qPCR data were statistically analysed using permutational multivariate analysis of variance (PERMANOVA) using the adonis2 function (200,000 permutations, sequential terms model) from the vegan package. First, the model specification $Y =Pen+Treatment + Time + Treatment:Time$ was used for the full timeseries and then the model specification $Y =Pen+ Treatment$ was used for the individual dates. The variance explained by the Treatment and/or Time, e.g. the R^2^-value, was used as the main metric for quantifying the effect of treatment on the microbiome and resistome. Pen was included as the first variable in all models to adjust the data for the variation stemming from pens and stables. The effect of sow was not adjusted for due to the standard practice of frequent mixing of piglets between sows and the low number of pigs sampled per sow. The data was correspondingly visualized with non-metric multidimensional scaling (nMDS). The β-dispersion, a measure of multivariate variance within groups, was calculated with the betadisper() function.

The α-diversity was calculated as the Shannon-index and analysed with a linear model specified as $Y = Treatment + Time + Treatment:Time$, and post-hoc tests were conducted as tests of marginal means of Treatment at each time point.

For univariate analysis of 16S rRNA gene data, ANCOMB-BC [29] with standard settings was used to find differentially abundant bacteria (for both ASVs and genera) at each time point between RWA and treated pigs. For univariate analysis of ARG data, the overall load of resistance genes was calculated as the sum of gene abundance and shown as medians. For individual genes, the analysis was further extended to include the time-of-treatment rather than treatment status only. Since the ARG data was highly heteroscedastic and zero-inflated, all ARG testing was conducted using the non-parametric Kruskal–Wallis test followed by Conovers test against the control at each time point. Genes were only tested if at least one time-of-treatment group had more than 50% non-zero values.

In order to link bacterial genera to ARGs, network analysis was carried out on separate time points and considering animals treated/untreated with the netComi-package using spearman correlations and the clr-transformation [39]. Specifically, the diffnet-function was used to calculate differentially important nodes between treated and untreated samples through their eigenvalue centrality. Resistance genes having high differential importance where then re-inspected for high differential correlations, and these observations were confirmed in metagenomics Hi-C data, if possible, by blastn search of ARG gene amplicons against the metagenome-assembled genomes (MAGs).

*P*-values were adjusted within datasets using the Benjamini–Hochberg procedure.* P*-values below 0.05 were considered significant.

## Results

In this study we investigated a batch of 513 ear-tagged pigs from an integrated RWA farm, of which we followed 202 pigs in detail for 5 months and investigated their faecal microbiome and corresponding antimicrobial resistance gene (ARG) profile.

### The microbiome diverges after treatment but converges over time

**Fig. 2:**
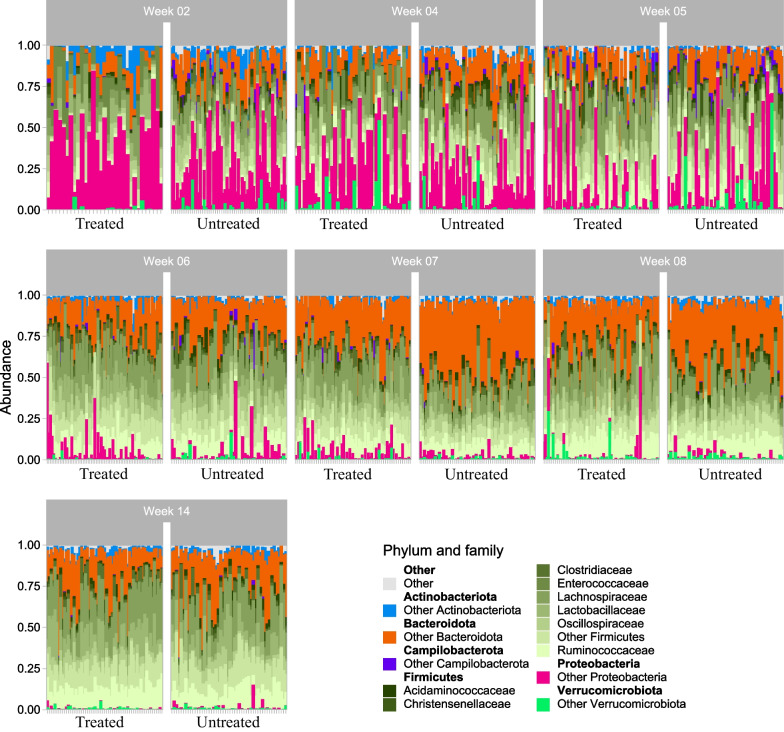
The distribution of key taxa observed in the study, stratified on major phyla and families. The microbiome of each sample was evaluated by sequencing of the V3V4 region of the 16S rRNA gene

To investigate the overall effect of antibiotics on the microbiome composition, all samples were analysed with 16S rRNA metataxonomics. Overall, the microbiome was dominated by the phyla *Firmicutes*, *Bacteroidetes, Proteobacteria* and *Actinobacteriota*. While *Proteobacteria* were particularly dominant in the early stages of the study, this phylum was eventually outcompeted in favour of members of *Firmicutes* and *Bacteroidetes* (Fig. 2).

**Fig. 3:**
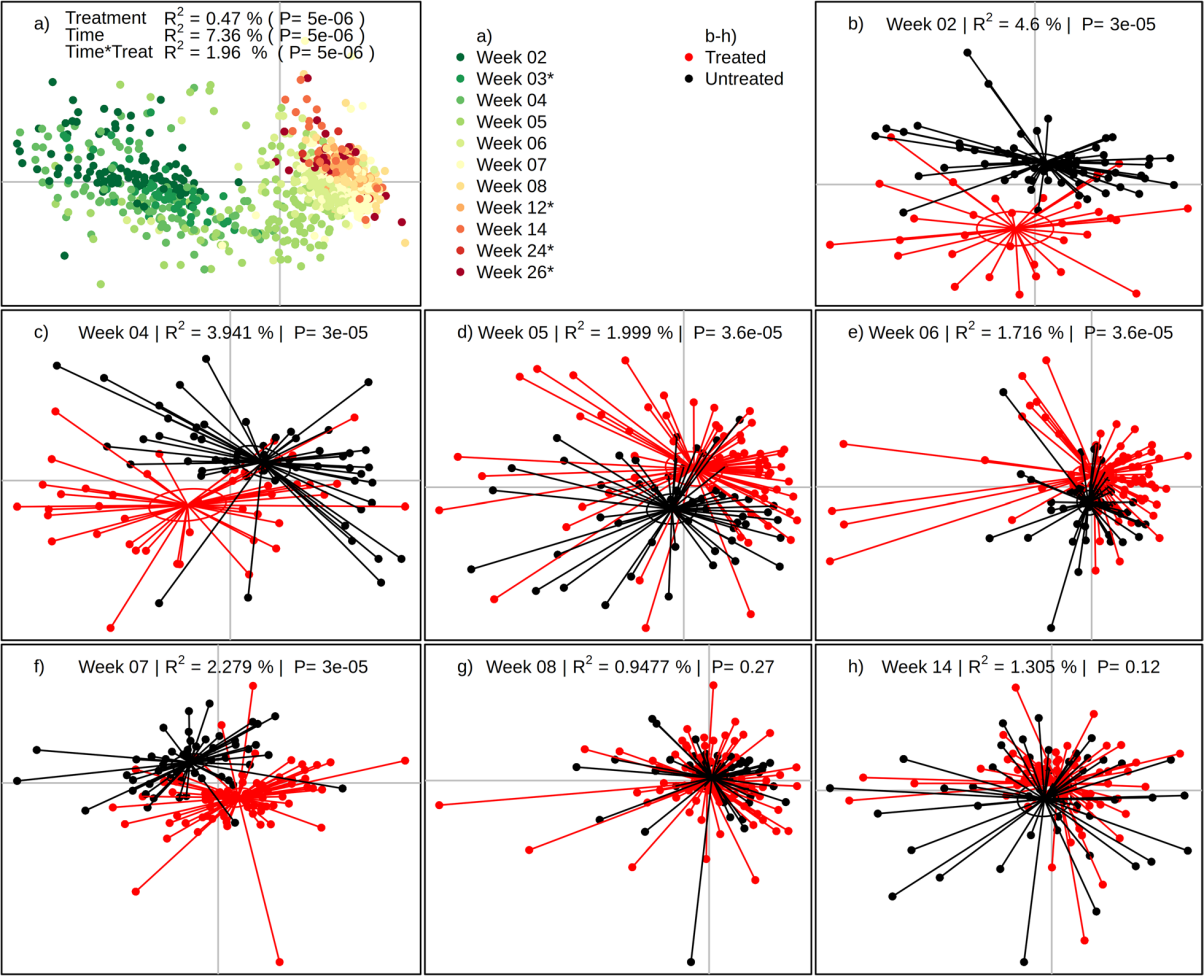
The effect of antibiotics on the porcine gut microbiome. The microbiome of each sample was evaluated by sequencing of the V3V4 region of the rRNA gene. **a** All samples of the study coloured by sampling time. Weeks marked with * in the legend are not included in the other panels due to under-sampling. **b**–**h** Animals were stratified at each time point into untreated (black) and antibiotic treated groups (red). Significance was evaluated by PERMANOVA at each time point adjusting for pen and variance described by treatment status is represented by the R^2^-value and corresponding p-value. Ellipses represents the 95% standard error of the centroids of each group. All panes are represented by the first and second nMDS-axis. Since the axes of nMDS are arbitrary, they are removed for brevity

First, the multivariate effect of treatment status along the sampling period was analysed with PERMANOVA whilst taking into account the individual pen of the pigs. Across all sampling points, the main driver of the taxonomic composition was the sampling week (R^2^ = 7.36%, *p* = 5 × 10^–6^) and to a substantially lesser extent the treatment status (R^2^ = 0.47% *p* = 5 × 10^–6^) as well as the interaction term of treatment and time (R^2^ = 1.96% *p* = 5 × 10^–6^) (Fig. 3a). Interestingly, the data show a clear clustering into two groups prior (weeks 2–4) and post (weeks 6–14) weaning, whilst the samples from week 5 (immediately post-weaning) were present in both clusters. As a significant interaction of treatment and time was observed, we proceeded to analyse each time point separately to elucidate the temporal details of metataxonomic dynamics of antibiotic treatment.

At the initial sampling time (week 2), 55 animals had been treated at least once, resulting in a significant divergence in β-diversity between treated and untreated animals (Fig. 3b,  R^2^ = 4.60%, *p* = 3 × 10^–5^). At weeks 4, 5, 6 and 7 this divergence progressively decreased and disappeared entirely at week 8 and 14 (Fig. 3b–h) as evident by decreasing R^2^ values (3.94%, 1.99%, 1.72%, 2.28%, 0.95%, 1.31%, respectively), suggesting that the initial perturbation of the microbiome was sustained for at least 7 weeks, but that the microbiome composition of treated and untreated animals have converged at week 14.

The α-diversity, as calculated by the Shannon index, was increased in both treated and untreated animals over time and appeared to stabilize at week 6 and beyond (Additional file 1: Figure S2). Between treatment groups, however, the α-diversity was lower in the treated pigs as compared to untreated pigs at week 2 and 4 by 18% and 11%, respectively, but was unaffected by treatment in the remaining weeks.

To analyse individual ASVs, we took advantage of the ANCOMB-BC pipeline [29], which allows for significance testing of differential abundance in compositional data. When performing this analysis, we focused on ASVs that were consistently (e.g. at 2 time points or more) differentially abundant between treated and untreated animals. With this approach, we found 14 ASVs with differential abundances of which 9 were higher in treated animals (members of *Erysipelotrichaceae, Enterococcaceae, Lachnospiraceae, Oscillospiraceae* and *Anaerovoracaceae*) and 5 were lower (members of *Tannerellaceae, Streptococcaceae, Christensenellaceae, Fusobacteriaceae* and *Chlamydiaceae*) (Additional file 1: Table S3). Although only significantly different in week 2, a high level of an ASV belonging to the genus *Escherichia-Shigella* was elevated in the treated group as well. As our method of sequencing the V3V4 region of the 16S rRNA gene rarely allows differentiation into species or strains, we repeated this analysis at the genus level and found 48 genera to be differentially abundant, likely due to spurious ASVs and species now being collected into genera, thus decreasing variance. The genera with higher abundance in the treated animals were exclusively various members of the *Clostridia* and *Bacillus*, while the genera lowered in treated animals were much more diverse constituting 26 genera in 7 different classes (Additional file 1: Table S4). Of particular interest are the genera *Prevotella* and *Desulfovibrio*, both of which being the only genera with higher differential abundance in untreated animals across 3 weeks. Almost all of the differentials, be it ASVs or genera, were different at weeks 2 and 4.

### The resistome diverges more than the microbiome after treatment and takes longer to converge

**Fig. 4:**
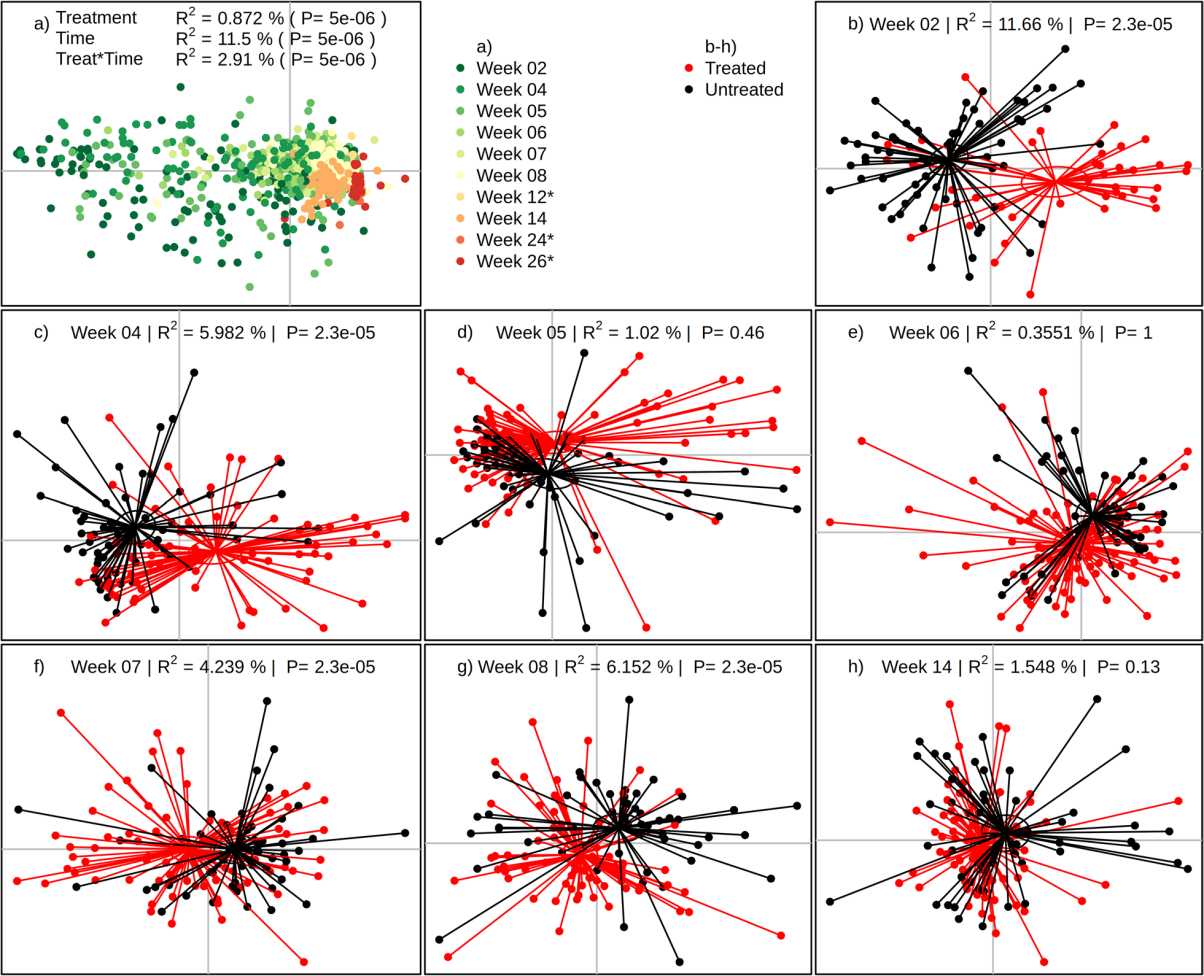
The effect of antibiotics on the porcine gut resistome. The resistome of each sample was evaluated by high-throughput qPCR on 82 ARGs. **a** All samples of the study coloured by sampling time. Weeks marked with * in the legend are not included in the other panels due to undersampling. **b**–**h** Animals were stratified at each time point into untreated (black) and antibiotic treated groups (red). Significance was evaluated by PERMANOVA at each time point adjusting for pen and variance described by treatment status is represented by the R^2^-value and corresponding p-value. Ellipses represents the 95% standard error of the centroids of each group. All panes are represented by the first and second nMDS-axis. Since the axes of nMDS are arbitrary, they are removed for brevity

We used a high throughput qPCR array to estimate the abundance of 82 antibiotic resistance genes, of which 22 were not detected in any sample and an additional 16 were only detected in 50 or less samples. The largest effect observed was from Time (R^2^ = 11.5%, *p* = 5 × 10^–5^), suggesting that age or time-since-treatment was the main driver of resistome β-diversity, while treatment (R^2^ = 0.87%, *p* = 5 × 10^–5^) and treatment:time interactions (R^2^ = 2.91%, *p* = 5 × 10^–5^) were of lesser effect (Fig. 4a). Nonetheless, analysis of individual sampling times revealed time-specific effects of treatment: At week 2 (Fig. 4b), the variance explained by Treatment status (R^2^ = 11.66%, *p* = 2 × 10^–5^) was considerably (2.5x) higher than the effect size found in the microbiome at 4.60%. However, a convergence at the time of weaning in weeks 5 and 6 was observed in the resistome (R^2^ = 1.02% and 0.35%, respectively), which, in contrast to the microbiome data, was not an effect of increased β-dispersion due to weaning and inclusion of zinc oxide (Additional file 1: Figure S3). In contrast to the taxonomic data, however, the groups diverged again at weeks 7 and 8 (R^2^ = 4.24% and 6.15%, respectively) followed by convergence at week 14.

### Analysis of multiple farms shows no effect of antibiotic treatment on the microbiome and resistome at the time of slaughter

**Fig. 5:**
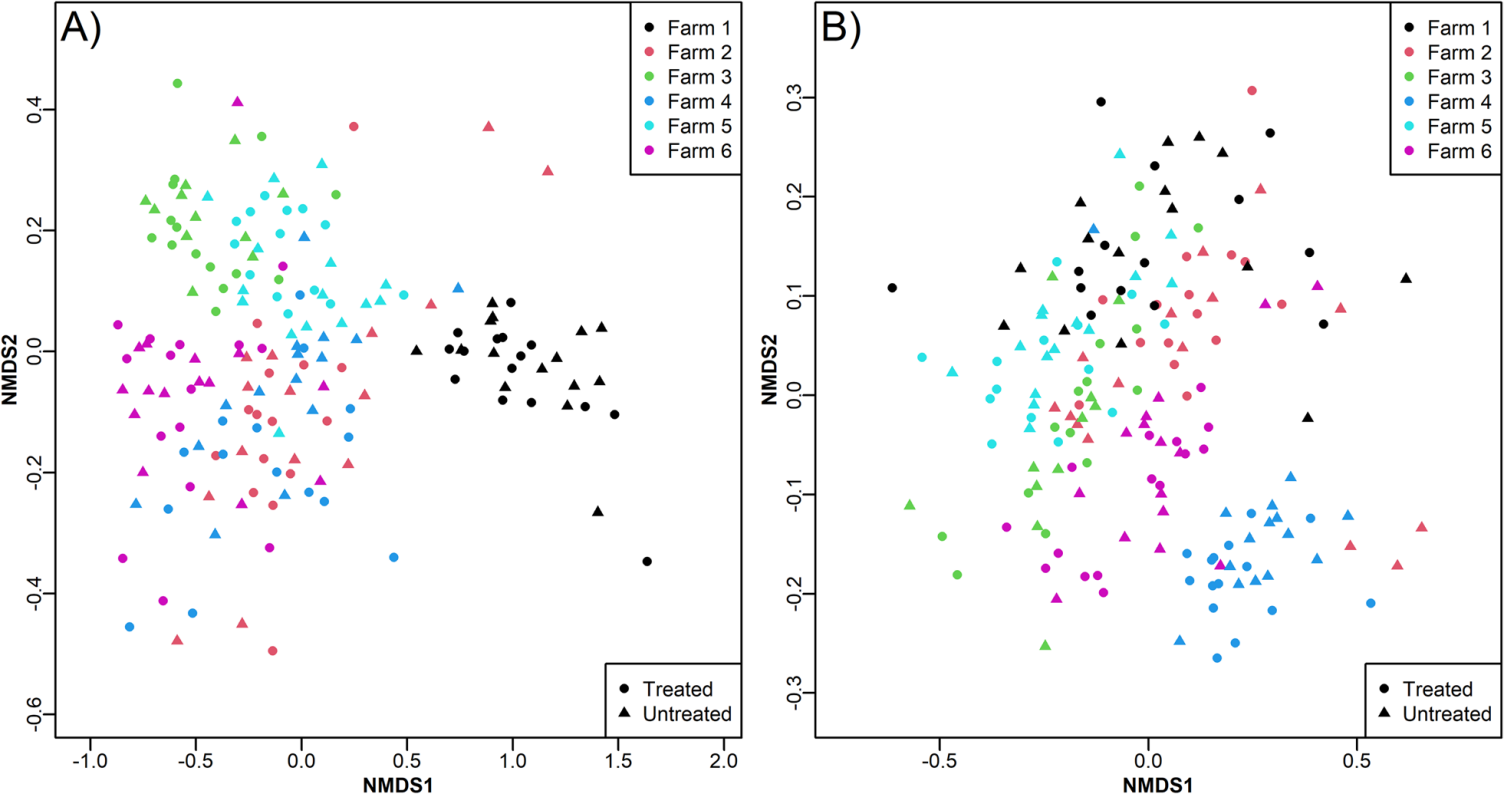
The effect of antibiotic treatment on a set of 176 pigs from 6 different farms taken at termination (26 weeks) at a slaughterhouse. **A** The microbiome of each sample as evaluated by sequencing of the V3V4 region of the rRNA gene. **B** The resistome of each sample as evaluated by high-throughput qPCR on 82 ARGs. Significance was evaluated by PERMANOVA and showed a significant effect of farm, but not of treatment status

Although samples close to slaughter (~ 26 weeks) are particularly relevant for the consumer, data from weeks 24 and 26 had to be excluded due to uneven and missing sampling (see Materials and Methods). Instead, we included additional slaughterhouse samples from 176 pigs from 6 different RWA farms (n_treated_ = 11–15 and n_untreated_ = 14–15 per farm). Analysis of these samples showed a significant effect of individual farm (p = 2 × 10^–5^) on the composition of both the microbiome and resistome (Fig. 5), but no effect of treatment status nor the interaction of the two. This data substantiated the findings in the cohort study, e.g., that the microbiomes and resistomes of treated and untreated animals converged over time, but also highlights that microbial composition is likely farm specific.

### Resistance genes abundance corresponds to time-of-treatment

**Fig. 6:**
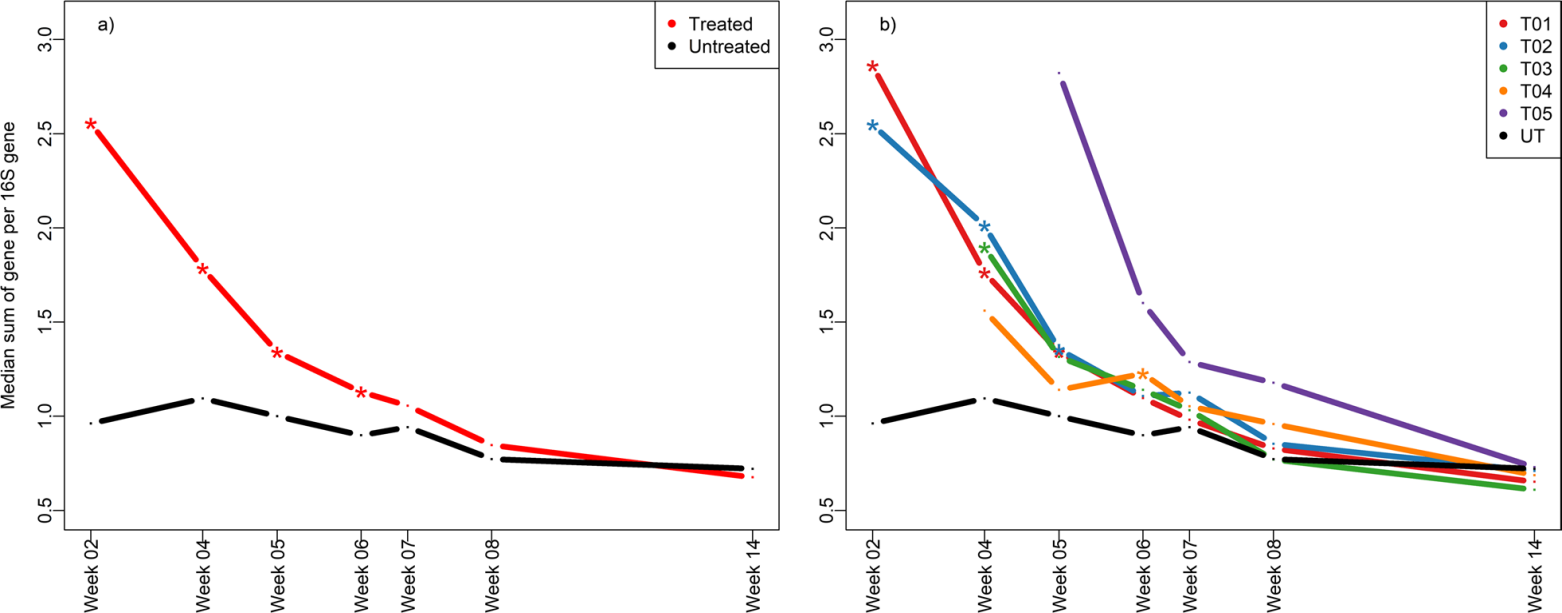
Resistance gene abundance across the study. The overall abundance of ARGs presented as the median sum of resistance genes in each pig per sampling time. **a** Samples are considered treated or untreated and **b** samples are considered on a time-of-treatment basis. Significant difference between treatment group and control at sampling time by Kruskal–Wallis test followed by Conover’s test is denoted by * at each time point. T01-T05 denotes treatment at the corresponding week. UT: untreated

Since substantial effects from antibiotic treatment was observed on the overall composition of the resistome at most time points, the individual genes responsible for this difference were investigated. Firstly, the total sum of ARG abundance were higher in the treated pigs compared to untreated pigs in week 2, 4, 5 and 6, but not in weeks 7, 8 and 14 (Fig. 6a), suggesting that the treatment with antibiotics increases the overall load of ARGs. Importantly, when this analysis is extended to include the time-of-treatment, the increase in ARG abundance appears to be matching the treatment times as well, e.g., time-of-treatment is followed by an abrupt increase in ARG abundance relative to untreated animals for animals treated in weeks 1, 2 and 3, but non-significantly in animals treated in week 4 and 5, presumably due to low numbers (Fig. 6b).

Detailed analysis of individual genes revealed that 25 of the 82 genes were differentially abundant from the control at one time-point or more (Additional file 1: Figure S4). Specifically, the genes *aacA-aphD* (gentamicin resistance), *ermT* (erythromycin (macrolide) resistance), *lnuB* (lincosamide resistance), *strB* (streptomycin resistance), *sul2* (sulfonamide resistance), *tetA* (tetracycline resistance), *tetM* (tetracycline resistance) and *blaTEM* (β-lactam resistance) were increased in abundance in treated pigs in concordance with time-of-treatment. In contrast, *tetB* and *tetC* (both tetracycline resistance) were lowered at weeks 2 and 4 in animals treated in weeks 1 and 2, mainly due to control values being initially high and later approaching zero around weaning. Of special interest was the mobile-element family of genes, of which *IS26* (insertion sequence), *IS1216* (insertion sequence), IS6100 (insertion element) and *IncN rep* (plasmid replication gene) were strongly affected. Although the levels of the *IncN rep* gene in control animals was fairly constant, this gene exhibited dynamic abundance in treated animals and was increased according to day-of-treatment before weaning, but showed a notable post-weaning effect only in treated animals. *czrC1*, a zinc resistance gene, was undetectable in most samples, and appeared to not be affected by zinc treatment.

### Resistance genes may be transferred by plasmids

**Fig. 7:**
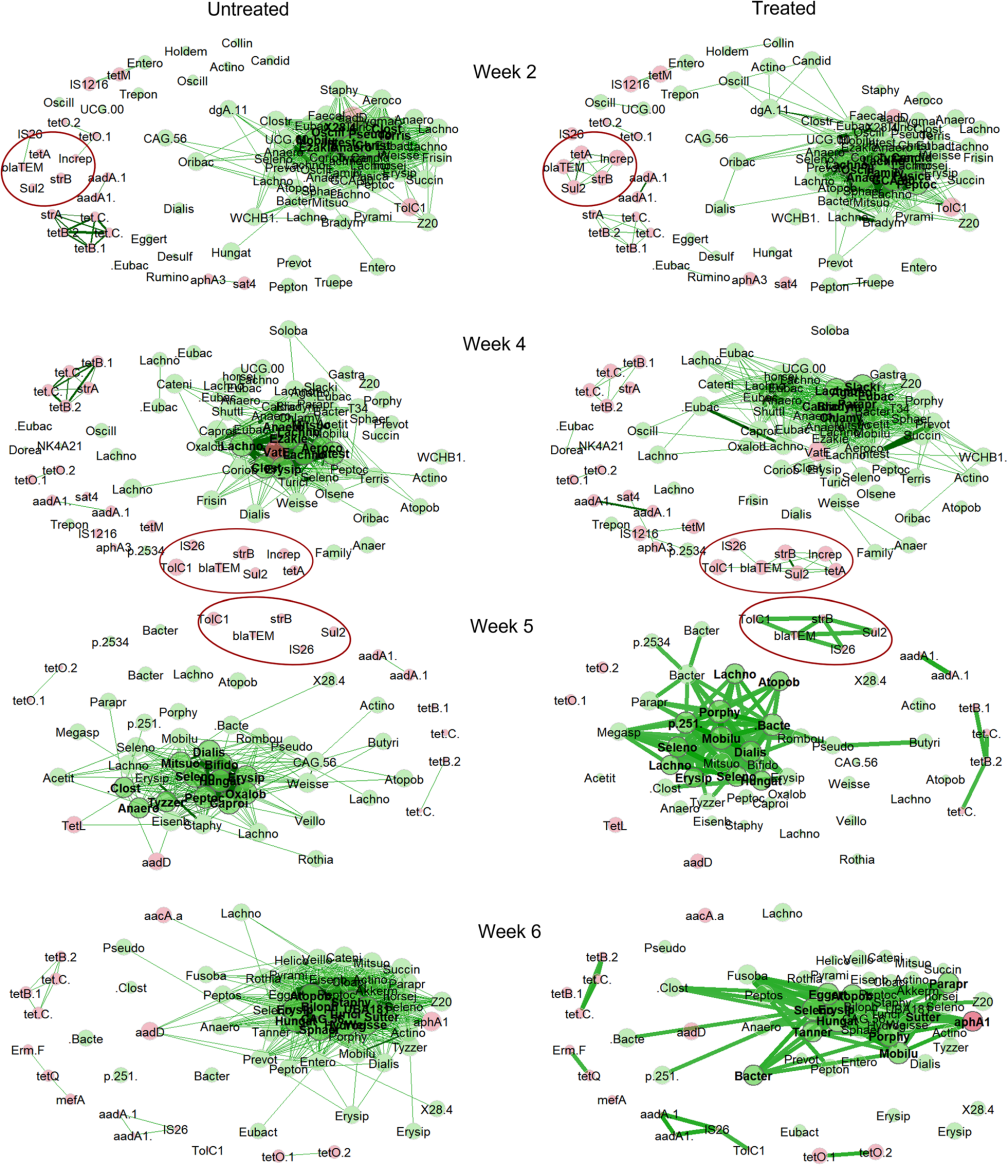
Co-occurrence networks of bacterial and ARG abundance for untreated and treated animals at week 2, 4, 5 and 6, highlighting how genes and bacteria associate differently depending on treatment. Networks were generated using centered log-transform normalization and a correlation cut-off of 0.7. The *IncN rep/IS26* cluster of differentially abundant genes is highlighted by red circles. Edges are scaled according to correlation

Since a larger effect on the resistome (Fig. 4) than the microbiome (Fig. 3) was observed upon antibiotic treatment and we observed a high differential abundance of plasmid and transposon genes, it can be speculated that resistance genes are mainly transferred on mobile elements rather than within individual bacterial taxa. In order to investigate this hypothesis, the resistome and metataxonomic data were collectively subjected to a co-occurrence analysis to elucidate which bacteria contain ARGs and more importantly to calculate evidence for ARGs being transferred by mobile elements (Fig. 7). In this approach, we saw a strong and consistent co-occurrence of *strA* and variants of *tetB/C* genes, which remained throughout the study. As a direct effect of antibiotic treatment, however, a strong differential association between a cluster of the genes *IncN rep*, *tetA*, *strB*, *sul2* and *blaTEM* was consistently observed in treated animals in weeks 2, 4 and but not in week 5 and 6; this was not observed in the untreated animals. Interestingly, the *IncN rep* gene of this cluster appeared to have been replaced by *IS26*, an insertion element, in week 5. Several other differential correlations between ARGs were observed, but none as strong as this cluster (Fig. 7).

Using differential network analysis on samples from treated vs. untreated pigs on each date, several genes and genera were observed to have significantly different roles between the networks as evident by the eigenvalue centrality metric, e.g. the importance of each node (ARG or bacteria). Again, the gene with the highest differential eigenvector centrality was the *IncN rep* gene, which upon treatment went from weakly (spearmans ρ < 0.3) to highly associated (spearmans ρ > 0.9) with *tetA*, *sul2*, *strB* and *blaTEM*. Inversely, *tetO.1* and *tetO.2* genes went from ρ > 0.8 to ρ < -0.1, suggesting that antibiotic treatment creates an evolutionary pressure on *IncN rep* plasmids which selects for *tetA*, *sul2*, *strB* and *blaTEM*. Analogous with these, a minor increase in correlation was observed with several bacteria that were uncorrelated in untreated samples.

To confirm the association between these ARG genes with other genes as well as various bacterial genera, we performed Hi-C metagenomics on a small subset of samples. Although the general aim of metagenomics analysis is to rebuild metagenome-assembled genomes using read coverage, GC-content and co-occurrence of sequences-by-sample, this approach cannot possibly connect chromosomes to plasmids as none of the above mentioned metrics are relevant for plasmids and their host chromosomes. Alternatively, Hi-C sequencing physically links DNA (chromosomal and plasmid) before sequencing and uses this information when building MAGs, thus being a method of additional interest for our data.

We then searched for the *IncN rep* gene in the resulting MAGs and found a perfect hit in a contig belonging to a MAG classified as *Shigella flexneri.* As *Shigella* spp. generally are associated with primates, even though *Shigella* spp. have been found to be able to infect other animals when introduced in animal experiments [15], in this study we find it more likely to be an *E. coli* found in metagenomes, as the two genera are difficult to distinguish [1, 44].

This MAG, moreover, was positive for 35 different ARGs, including the *tetA*, *sul2* and *blaTEM* genes, although not in the same contig as *IncN rep*. Unfortunately, the resolution of the V3V4 region of the 16S rRNA gene does not allow species resolution in most bacteria, much less the *Enterobacteriaceae* family [47], which prevented us from confirming these observations in our metataxonomic data.

In contrast to the *IncN rep* gene, which was only found in one contig, the *IS26* gene was found in 5 contigs and the *IS1216* gene was found in more than 500, making detailed analysis difficult.

## Discussion

In this study, the effect of antibiotic treatment on the faecal pig microbiome and resistome of individual pigs in a commercial farm setting was evaluated by microbiome metataxonomics and high-capacity quantitative PCR arrays combined with metagenomic sequencing.

### The resistome is affected by antibiotic treatment for longer time than the microbiome

We used PERMANOVA to evaluate the effect of antibiotic treatment on both the microbiomes and the resistomes, and observed the highest variance was explained by the treatment status at week 2 relative to later time points. Although it is difficult to generalize disease patterns across different farms, the farm investigated in this study had an unusual timing in terms of onset of disease, e.g. in the farrowing unit at weeks 2 and 4, whilst most other farms report post-weaning diarrhoea as being the major cause of disease and, hence, treatment [40]. In contrast, the majority of the animals in our study were treated in weeks 1, 2, 3 and 4. This may limit whether or not our observations can be extended nationally or globally. We did investigate the external validity through the analysis of 6 different farms which revealed a considerable farm specific effect (Fig. 5). Despite this, we observed general convergence of treated and untreated animals on our cohort as well as in the end-point data, suggesting that individual farms may have separate dynamics, but the effect of antibiotics is likely to have disappeared at the time of slaughter. In the weeks following treatment, a steady decrease in R^2^ from treatment was observed in the microbiomes of treated and untreated pigs. At week 8 (Fig. 3) we did not observe a significant difference between treated and untreated animals, which suggest the microbiomes of the treated pigs have recovered at the latest 8 weeks having been treated in weeks 1, 2, 3 and 4. Our results concur with a previous study showing divergence of the microbiome between control pigs and pigs treated systemically with oxytetracycline, which also converged over time [41]. Since pigs are coprophagic, they will share their microbiomes and this is a probable reason for the observed convergence. We are currently investigating this in controlled settings. As previously seen, the analysis also showed that untreated pigs have a more diverse microbiome than the pigs treated with antibiotics [20, 43, 52]. Conversely, Looft et al. [31] found that both abundance and diversity of antibiotic resistance genes increased in medicated pigs compared to unmedicated, even in pigs with a high background of resistance genes.

As was seen in the microbiome, the resistome showed an altered composition in animals treated with antibiotics, although to a markedly greater degree as evident by the treatment-explained variance at week 2 being 11.7% compared to 4.6% in the microbiome (Fig. 4). This effect continued to be evident up until week 8, although there appears to be a substantial effect of weaning in weeks 5 and 6, which independently disturbs the resistome and masks the effect of antibiotic treatment. Although few pigs in this study were treated for post-weaning diarrhoea, we observed a substantial disruption of the microbiome, as also described by Karasova et al. [27], when piglets were removed from the sow and plant-based was introduced (Additional file 1: Figure S3). This antibiotic treatment effect was consistent until 14 weeks, however, the pigs were continuously moved to the finishing unit along week 12 since the study was conducted on a commercial farm, and it cannot be ruled out that this movement had an effect on the resistome. Analogous to the microbiome, a treatment effect on the resistome was not evident in the slaughterhouse samples, as the samples also here showed a strong farm effect. Individual farms will have distinct microbial profiles and one should be careful in generalizing across farms.

The observation that the treatment effect is stronger in the resistome than the microbiome suggests that disturbance of the resistance genes is decoupled from the overall taxonomic composition, either by subtle increases in highly resistant bacteria or by increase in plasmids carrying ARGs, either through heightened copy number or transfer to other bacteria. We observed an increase in ARG load corresponding to treatment status as well as a systematic increase according to time-of-treatment (Fig. 6), suggesting that ARG abundance is a direct function of treatment. Although the resistance load decreases when the treated animal moves away from time-of-treatment, the initial increase in resistance gene abundance is still of concern as this spike will contribute to the overall load on the farm, and hence to society at large.

### The microbiome and resistome of treated and untreated pigs converges

The experimental design of this study does not allow us to determine if the convergence observed between treated and untreated pigs in both the microbiome and the resistome is due to the microbiome of the individual pig returning to a ‘standard’ microflora or is the result of mixing of treated pigs and untreated RWA pigs. As pigs are coprophagic, it can be speculated that it could also be a result of transfer of bacteria and their resistance genes resulting at an equilibrium after some time.

### Several genera are significantly different in abundance between groups, most found in the first weeks

In order to elucidate which bacterial genera are significantly different in abundance between treated and untreated pigs and hence are drivers of the divergence between the groups, we used ANCOM-BC which is designed to handle relative abundances. We observed relatively few ASVs as being differentially abundant at more than two time points, which may be due to technical limitations of our metataxonomic method [47]. An ASV belonging to the *Escherichia-Shigella* genus was, however, higher in treated animals but only in week 2. Repeating the analysis on the genus level revealed that the *Prevotella* genus was higher in untreated pigs in weeks 2, 4 and 7. *Prevotella* is a commensal genus in pigs and is generally associated with health, in terms of feed efficiency and pathogen resilience [14], although some species of *Prevotella* can also act as pathogens [4]. In our current study, the *Prevotella* genus was found to be more abundant after weaning, as has previously been observed [33], though with no significant difference between treated and untreated pigs after week 7. *Desulfovibrio* was significantly higher untreated animals in weeks 2, 4 and 7, and has previously been found in to be negatively correlated with average daily gain, backfat thickness, daily feed intake, feed conversion ratio and residual feed intake [3]. As for the members of the family *Erysipelotrichaceae,* which were elevated in treated animals, some species are highly antibiotic resistant as seen in human clinical isolates [2, 9]. The *Enterococcus* genus was also more abundant in the antibiotic treated pigs at week 2 and 4, and although this genus is generally considered a commensal bacteria in pig microbiomes, it is intrinsically highly resistant and has been highlighted as a potential reservoir of antimicrobial resistance which may be transferred to humans from pigs. *Enterococcus* is known to often harbour antibiotic resistance and infections with enterococci can therefore be difficult to treat [49].

Within the resistome data, 25 genes were significantly different compared to the controls in at least one time point, most of which in correspondence with the time-of-treatment. These genes were highly diverse in the types of resistance they encode, many of which are not used in routine farming such as *aacA-aphD* (gentamicin resistance), *ermT* (erythromycin resistance), *strB* (streptomycin resistance) and *sul2* (sulfonamide resistance), highlighting how antibiotic treatment will co-select for broad resistance as seen previously [31]. Curiously, the *tetB* and *tetC* were higher in the control relative to the treatment groups which is in contrast to the pattern of *tetA* and *tetM*, suggesting that these genes are inversely selected for, even though they all provide tetracycline resistance. However, we do not know whether these pigs had been treated with a tetracycline compound or another antibiotic. Such observations further add to the complexity of the associations between antibiotic exposure and development of resistance, which is not always simple and sometimes even inverse. Of further interest is the *tetX* gene, which increased dramatically after weaning only in animals having been previously treated, which may imply that previous treatment primes the microbiome for a certain response later on.

### Bacteria and gene association

In order to connect the microbiome and resistome data, we used network analysis to highlight which variables associated differently between treated and untreated animals. In weeks 2 and 4, the *IncN rep* gene, a plasmid marker gene, became highly associated with the *tetA*, sul2, *strB* and *blaTEM* genes in treated animals, suggesting that these resistance genes will be located on a plasmid under antibiotic pressure and can hence be shared more easily. The Inc-family of plasmids are widespread in *Enterobacteriaceae*, *Pseudomonas* and *Staphylococcus*, and the incN type in particular is a conjugative plasmid with broad host range within *Enterobacteriaceae* [42]. Analogous to our results, this type of plasmid usually carries a large amount of resistance genes, including *tetA*, sul2, *strB* and *blaTEM* genes [18, 42]. Since we did not see an equally strong association to any bacteria, it is likely that the plasmid resides in multiple different species of bacteria, as has also been proposed in an analysis of Chinese pig resistome using an approach similar to ours [25]. However, it is well known that the so-called ASSut (ampicillin-streptomycin-sulfonamide-tetracycline) resistance pattern, which is conferred by a combination of exactly these genes, is widespread in both *E. coli* [21] and *Salmonella* typhimurium [5] from pigs. Our approach of statistically linking resistance and microbial composition may hence both corroborate earlier data as well as suggest molecular mechanisms behind them. We next investigated Hi-C generated MAGs from a subset of samples and found the *IncN rep* gene in a contig belonging to *Shigella flexneri/E. coli*. As the MAGs are generated from several samples, and do not represent the entire time series, we cannot rule out that the observed correlation is merely due to presence of *Shigella flexneri/E. coli* although the low abundance of *Escherichia-Shigella* in week 4 suggests otherwise.

## Conclusion

We followed a cohort of 202 pigs from a commercial RWA farm across their lifetime in order to evaluate the response of their microbiome and resistome to antibiotic treatment. Here, we observed a divergence in both the microbiomes and resistomes between treated and untreated pigs according to antibiotic treatment and this persisted weeks after treatment. Both the microbiome and resistome did, however, recover before slaughter, perhaps since the treated pigs were not separated from the RWA pigs and they thereby shared their microbiome. Despite this convergence of the profiles of treated and untreated pigs over time, pigs treated with antibiotics excrete significantly more resistance genes following treatment, which may contribute to the pool of resistance genes in the broader society over their lifetime. These genes reflect, to some degree, the antibiotics used in standard treatments, although genes not directly relevant for treatment appear to be co-selected for. A high level of correlation between resistance genes and mobile elements was evident in treated animals only, suggesting that antibiotic treatment selects for plasmid-borne resistance genes. The results highlight the complex relations between antibiotic exposure and development of resistance. Further studies are needed to elucidate if physical separation of animals can further decrease the level of resistant bacteria in untreated animals.

### Supplementary Information

**Additional file 1.** Supplementary Figures and Tables.

## Data Availability

The datasets analysed during this study are available from the corresponding author on reasonable request. Sequence data is available at SRA as project SUB12915294. Code for analysis is available at https://github.com/katrinetams/Cohort_study_2021.
