# Supplementary material for: The effects of antibiotic use on the dynamics of the microbiome and resistome in pigs
Source: Anim Microbiome. 2023 Aug 21;5:39. doi: 10.1186/s42523-023-00258-4 (PMC10440943; doi:10.1186/s42523-023-00258-4)
Supplement: Supplementary file 1 — Additional file 1. Supplementary Figures and Tables. [file 42523_2023_258_MOESM1_ESM.docx]

Supplementary material

The effect of antibiotic use on the dynamics of the microbiome and resistome in pigs

Katrine Wegener Tams^1^, Inge Larsen^2^, Julie Elvekjær Hansen^1^, Henrik Spiegelhauer^1^, Alexander Damm Strøm-Hansen^1^, Sophia Rasmussen^1^, Anna Cäcilia Ingham^4^, Lajos Kalmar^3^, Iain Kean^3^, Øystein Angen^4^, Mark Holmes^3^, Karl Pedersen^5^, Lars Jelsbak^1^, Anders Folkesson^1^, Anders Rhod Larsen^4^ and Mikael Lenz Strube^1#^

^1^Technical university of Denmark, Kgs. Lyngby 2800, Denmark, ^2^University of Copenhagen, Copenhagen 1871, Denmark, ^3^University of Cambridge, Cambridge, United Kingdom, ^4^Department of Bacteria, Parasites and Fungi, Statens Serum Institut (SSI), Copenhagen 2300, Denmark, ^5^ National Veterinary Institute, 751 89 Uppsala, Sweden

#Corresponding author:

Mikael Lenz Strube ([milst@dtu.dk](mailto:milst@dtu.dk))


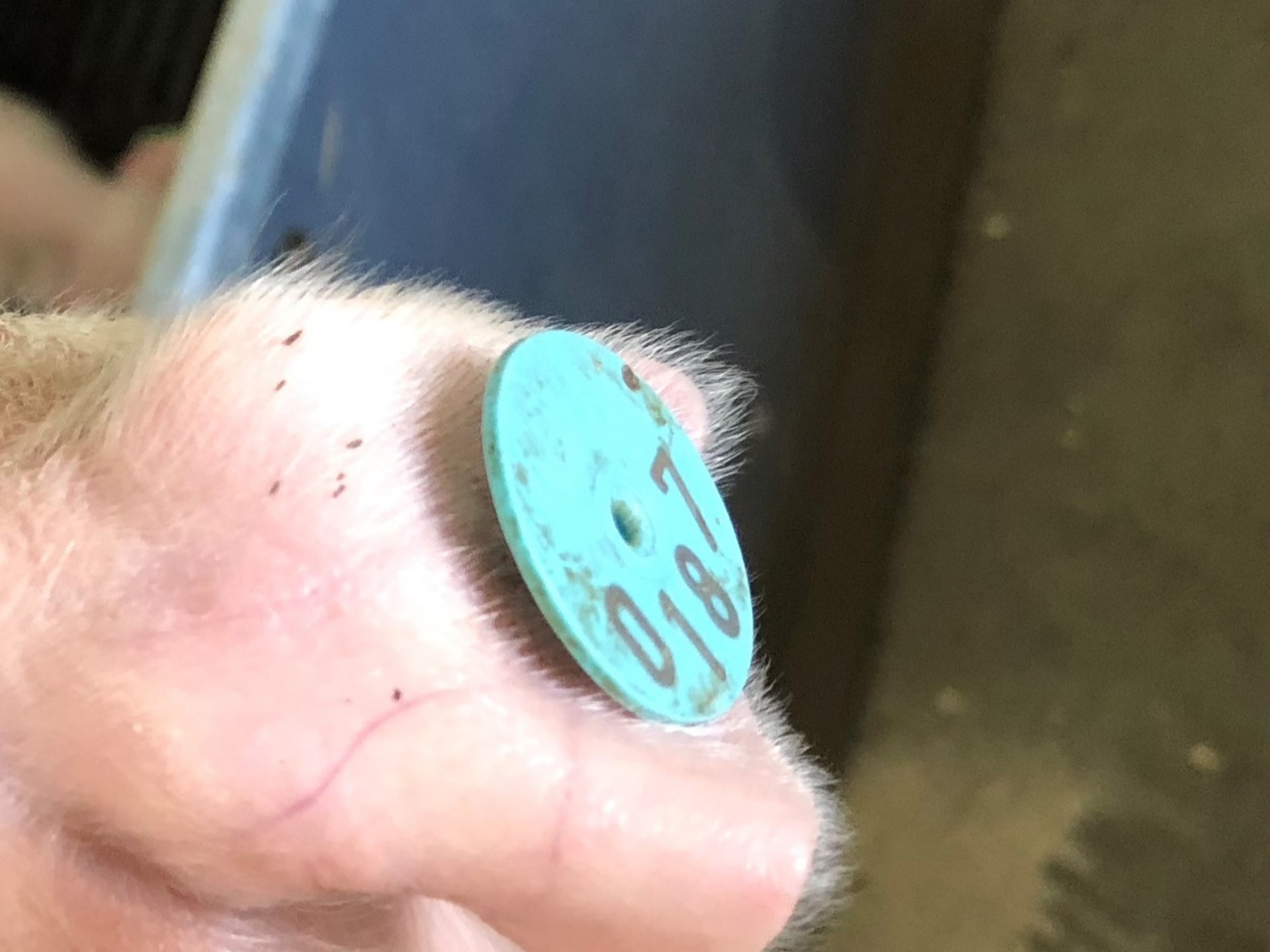


**Figure S1:** Ear tags used for marking RWA-pigs.


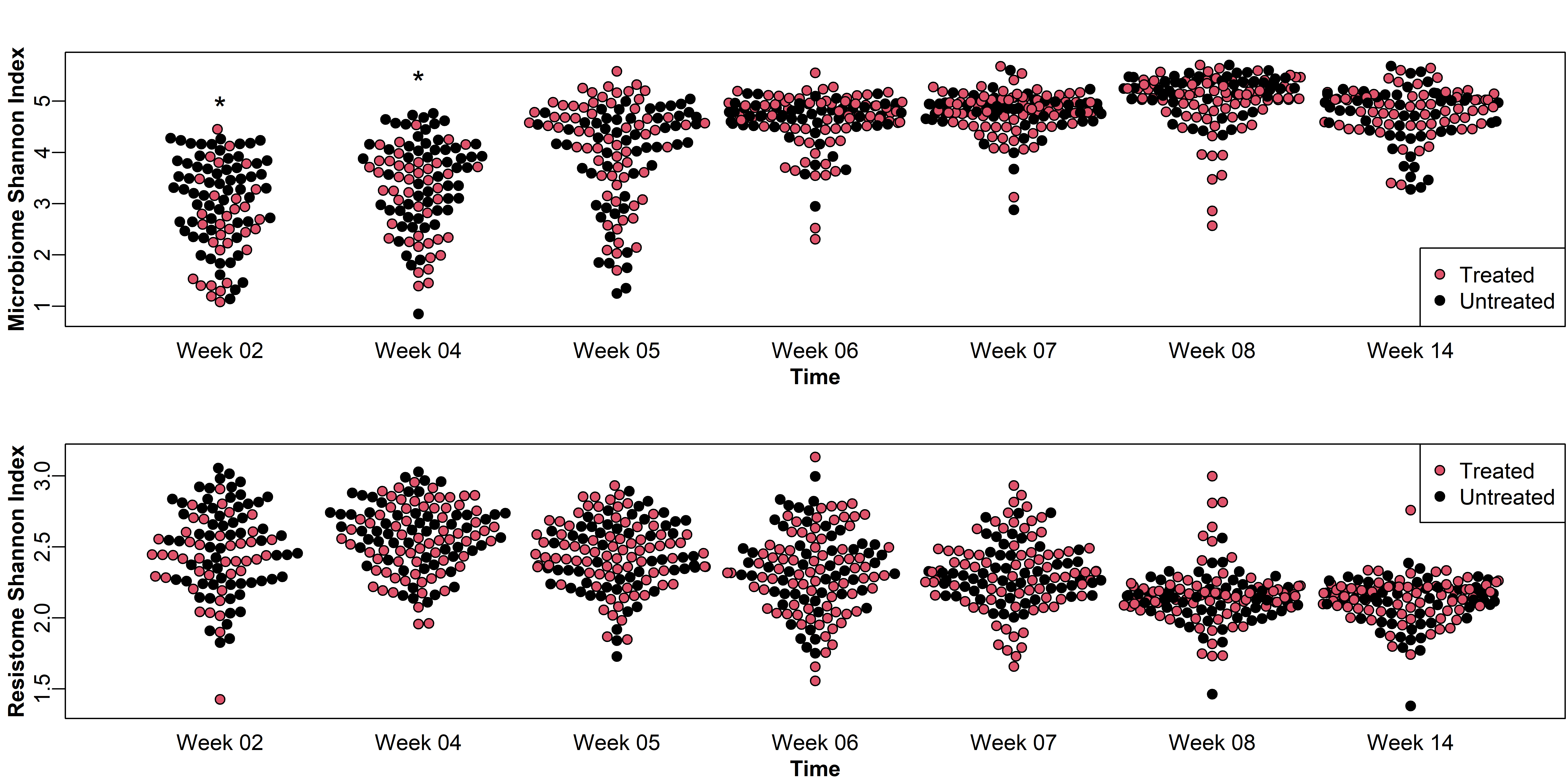


**Figure S2:** The alpha diversity as calculated by the Shannon index on the microbiome (top) and resistome (bottom) data. Data was analysed by linear models (Time + OUA + Time:OUA), and individual timepoints was further analysed by test of marginal means. *: significant difference between Treated and Untreated samples within timepoint.


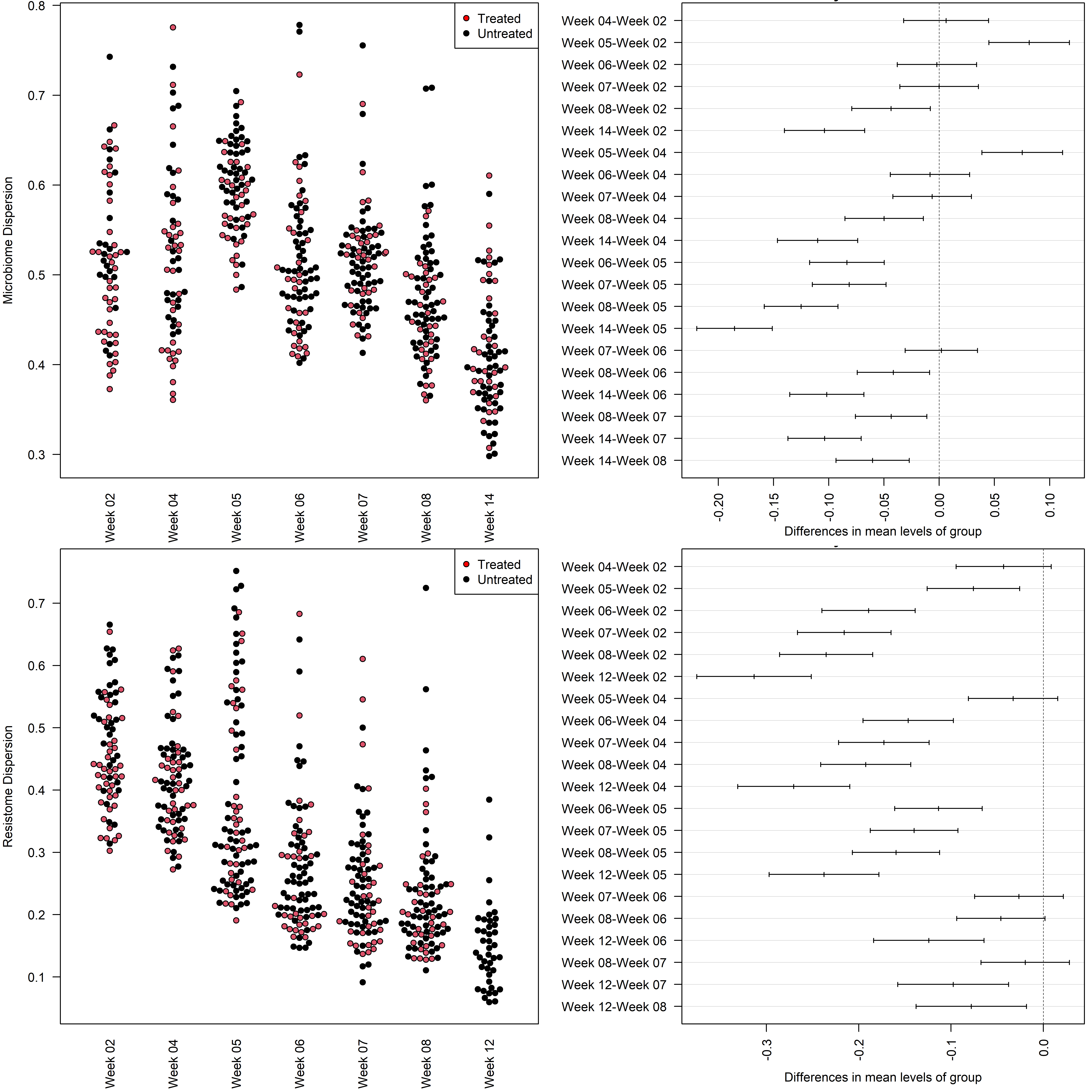


**Figure S3:** The beta-dispersion, e.g., the multivariate variance, of the microbiome (top) and resistome (bottom). Higher values correspond to more variability in the dataset. The right panels are pairwise comparisons of each timepoint, where confidence intervals not overlapping 0 are considered significant differences.


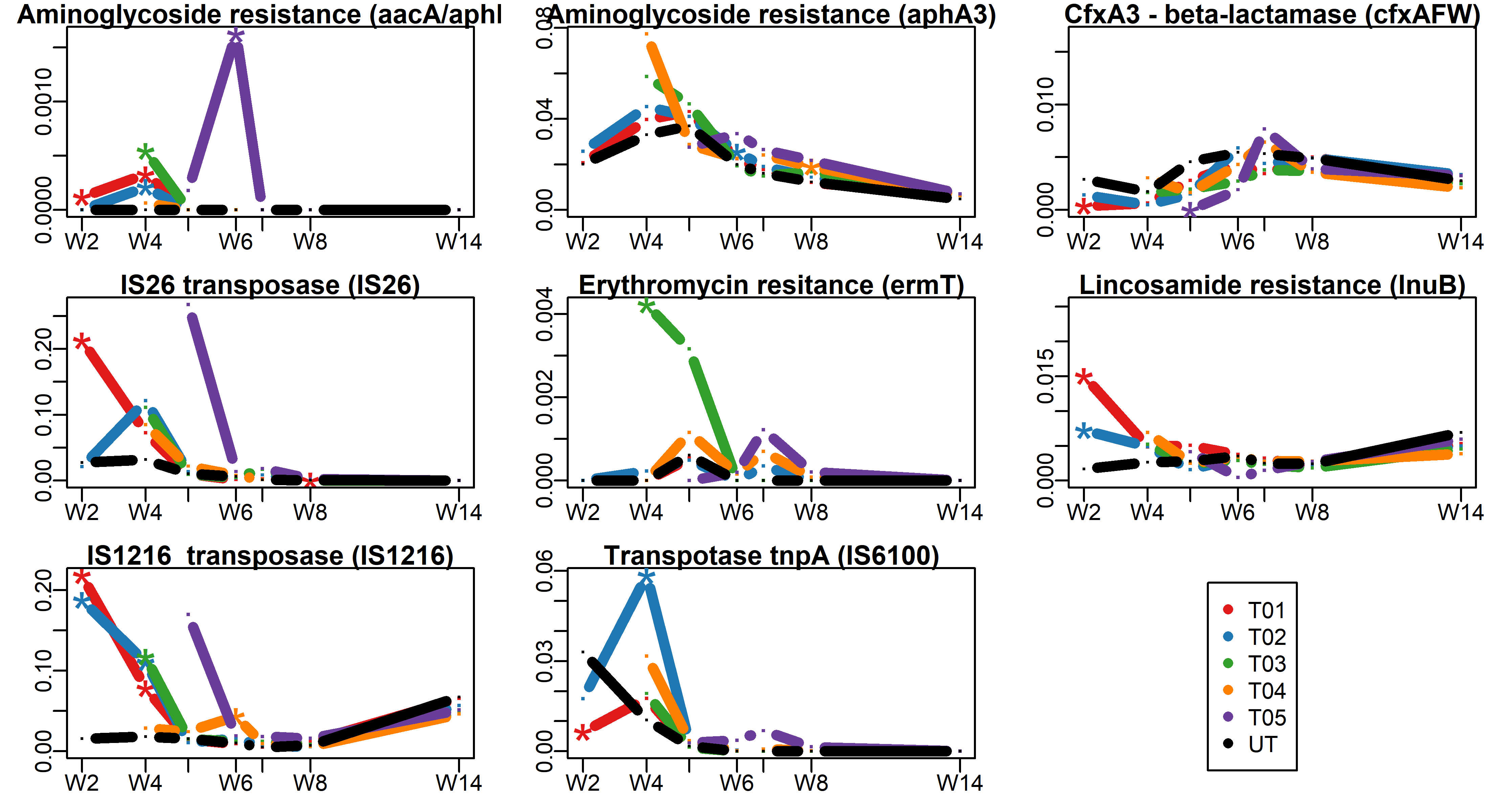

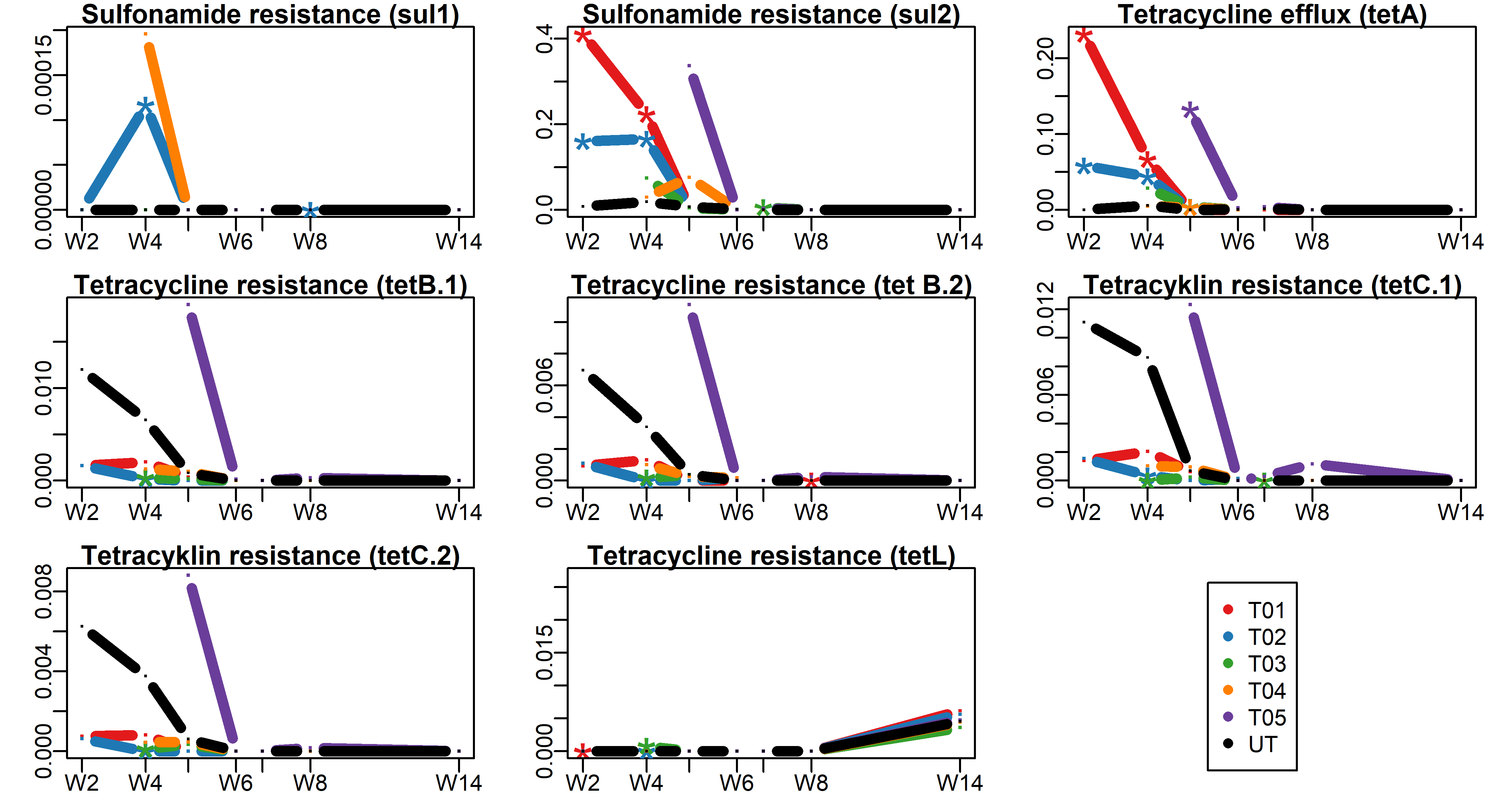

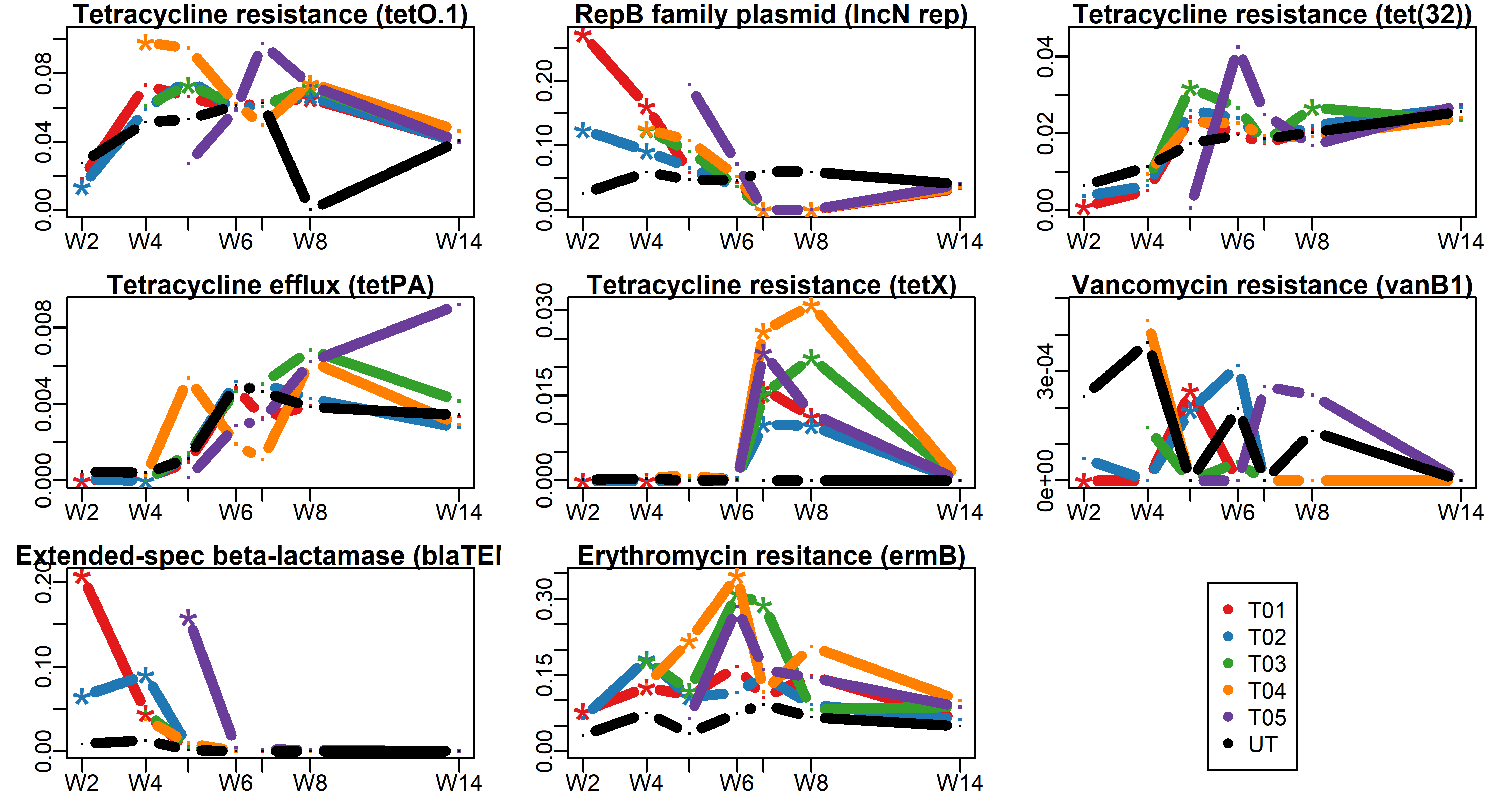


**Figure S4:** Individual resistance gene abundance across the study. The abundance of all genes with at least one time-of-treatment group different from the control on at least one timepoint. Significant difference between treatment group and control at sampling time by Kruskal-Wallis test followed by Conover’s test is denoted by * at each timepoint.

**Table S1:** Antibiotics used for treatment in this study.

| **Farrowing unit** | **Weaning Unit** |
| --- | --- |
| Sows: Sulfadiazine and Sulfadoxine (broad-spectrum sulfonamides) | Amoxicillin (a β-lactam antibiotic in the aminopenicillin group) |
| Sows: Benzylpenicillin (Penicillin G) (Benzylpenicillin-procaine) | Benzylpenicillin (Penicillin G) (Benzylpenicillin-procaine) |
| Piglets: Florfenicol (analog of thiamphenicol) | Dihydrostreptomycin (a derivative of streptomycin) |
| Piglets: Gamithromycin (macrolide) | Oxytetracycline |
|  | Gamithromycin |

**Table S2**: Target genes, primer sequences, amplicons, lengths and targets used for the high throughput qPCR platform.

| **Primer** | **FORWARD 5'end-3'end** | **REVERSE 5'end-3'end** | **amplicon** | | **bp** | **Gene/organism** |
| --- | --- | --- | --- | --- | --- | --- |
| *aacC4* | CGGCGTGGGACACGAT | AGGGAACCTTTGCCATCAACT | AGGGAACCTTTGCCATCAACTCGGCAAGATGCAGCGTCGTGTTGGCATCGTGTCCCACGCCG | | 62 | "aminoglycoside N-acetyltransferase AAC(3)-IV" : CP033224 : *Salmonella enterica* plasmid pCFSA 122-1 |
| *aadA 1- A (aadA zhup)* | GTTGTGCACGACGACATCATT | GGCTCGAAGATACCTGCAAGAA | GGCTCGAAGATACCTGCAAGAATGTCATTGCGCTGCCATTCTCCAAATTGCAGTTCGCGCTTAGCTGGATAACGCCACGGAATGATGTCGTCGTGCACAAC | | 101 | "ANT(3'')-Ia family aminoglycoside nucleotidyltransferase AadA1" : CP033224 : *Salmonella enterica* plasmid pCFSA 122-1 |
| *aadD* | CCGACAACATTTCTACCATCCTT | ACCGAAGCGCTCGTCGTATA | CCGACAACATTTCTACCATCCTTGACTGTACAGGTAGCAATGGCAGGTGCCATGTTGATTGGTCTGCATCATCGCATCTGTTATACGACGAGCGCTTCGGT | | 101 | Lactobacillus reuteri KLR1001 aadD1 gene for aminoglycoside O-nucleotidyltransferase ANT(4')-Ia : NG_056101 |
| *aph6ia* | CCCATCCCATGTGTAAGGAAA | GCCACCGCTTCTGCTGTAC | CCCATCCCATGTGTAAGGAAATTTCCCATGAGTTCGTCGGACCACATCCACGTCCCGGACGGCCTGGCCGAGTCGTACAGCAGAAGCGGTGGC | | 93 | "aminoglycoside O-phosphotransferase APH(6)-Ia" : NG_047458.1 : *Streptomyces griseus* N2-3-11 aph(6)-Ia gene for aminoglycoside O-phosphotransferase APH(6)-Ia |
| *aphA3* | AAAAGCCCGAAGAGGAACTTG | CATCTTTCACAAAGATGTTGCTGTCT | AAAAGCCCGAAGAGGAACTTGTCTTTTCCCACGGCGACCTGGGAGACAGCAACATCTTTGTGAAAGATG | | 69 | "aminoglycoside O-phosphotransferase APH(3')-IIIa" : CP033211.1 : *Enterococcus faecium* strain RBWH1 plasmid pRBWH1.5 |
| *blaCMY2b* | AAAGCCTCATGGGTGCATAAA | ATAGCTTTTGTTTGCCAGCATCA | AAAGCCTCATGGGTGCATAAAACGGGCTCCACTGGTGGATTTGGCAGCTACGTAGCCTTCGTTCCAGAAAAAAACCTTGGCATCGTGATGCTGGCAAACAAAAGCTAT | | 108 | "class C beta-lactamase CMY-2" : *Salmonella* *enterica* subsp. *enterica* serovar Newport str. CDC 2012K-0663 plasmid pSNE2-2012K-0663 : CP025245.1 |
| *blaCTX-M* | CACAGTTGGTGACGTGGCTTAA | CTCCGCTGCCGGTTTTATC | CACAGTTGGTGACGTGGCTTAAGGGCAATACTACCGGTAGCGCGAGCATTCGGGCGGGTCTGCCGAAATCATGGGTAGTGGGCGATAAAACCGGCAGCGGAG | | 102 | "extended spectrum beta-lactamase CTX-M" : *Escherichia coli* strain 1481 extended spectrum beta-lactamase CTX-M gene : MG581457.1 |
| *blaTEM* | AGCATCTTACGGATGGCATGA | TCCTCCGATCGTTGTCAGAAGT | TCCTCCGATCGTTGTCAGAAGTAAGTTGGCCGCAGTGTTATCACTCATGGTTATGGCAGCACTGCATAATTCTCTTACTGTCATGCCATCCGTAAGATGCT | | 101 | "class A broad-spectrum beta-lactamase TEM-1" : *Klebsiella pneumoniae* strain NH34 plasmid pNH34.1 : CP034406.1 |
| *blaIMP(2)* | AAGGCAGCATTTCCTCTCATTTT | GGATAGATCGAGAATTAAGCCACTCT | AAGGCAGCATTTCCTCTCATTTTCATAGCGACAGCACGGGCGGAATAGAGTGGCTTAATTCTCGATCTATCC | | 72 | "subclass B1 metallo-beta-lactamase IMP-80" : *Pseudomonas aeruginosa* NCGM 3336 *blaIMP* gene for subclass B1 metallo-beta-lactamase IMP-80 : NG_062274.1 |
| *cfiA* | GCAGCGTTGCTGGACACA | GTTCGGGATAAACGTGGTGACT | GCAGCGTTGCTGGACACACCGATCAATGACGCACAAACGGAAATGCTGGTCAACTGGGTGACAGACTCTTTGCATGCCAAAGTCACCACGTTTATCCCGAAC | | 102 | "CfiA-22 metallo-beta-lactamase" : *Bacteroides fragilis* *cfiA* gene for CfiA-22 metallo-beta-lactamase :LT714127 |
| *cfxA FW* | TCATTCCTCGTTCAAGTTTTCAGA | TGCAGCACCAAGAGGAGATGT | TGCAGCACCAAGAGGAGATGTATAGTTAGAGTAAGCCTTGTTATGGTCAGCCGACATTTCCTCTTCCGTATAAGCTATCTGAAAACTTGAACGAGGAATGA | | 101 | "CfxA3 - beta-lactamase" : *Capnocytophaga ochracea* plasmid pCAP01 MobA (mobA), CfxA3 (cfxA3), transposase-like protein, RepA (repA), relaxase-like protein, and mob-like protein genes : AY860640 |
| *dfrA12* | CCTCTACCGAACCGTCACACA | GCGACAGCGTTGAAACAACTAC | CCTCTACCGAACCGTCACACATTGGTAATCTCACGCCAAGCTAACTACCGCGCCACTGGCTGCGTAGTTGTTTCAACGCTGTCGC | | 85 | "trimethoprim-resistant dihydrofolate reductase DfrA12" *: Klebsiella pneumoniae* strain NH34 plasmid pNH34.1 : CP034406.1 |
| *lnuB* | TGAACATAATCCCCTCGTTTAAAGAT | TAATTGCCCTGTTTCATCGTAAATAA | TGAACATAATCCCCTCGTTTAAAGATTCAGGTTATATTCCTGATACGAAGGCTATGCTTATTTACGATGAAACAGGGCAATTA | | 83 | "lincosamide nucleotidyltransferase" : *Enterococcus faecalis* plasmid pKUB3007-1 KUB3007 DNA : AP018544.1 |
| *mecA* | GGTTACGGACAAGGTGAAATACTGAT | TGTCTTTTAATAAGTGAGGTGCGTTAATA | GGTTACGGACAAGGTGAAATACTGATTAACCCAGTACAGATCCTTTCAATCTATAGCGCATTAGAAAATAATGGCAATATTAACGCACCTCACTTATTAAAAGACA | | 106 | "penicillin binding protein PBP2A" : *Staphylococcus aureus* strain MRSA 422665 penicillin binding protein PBP2A (*mecA*) gene : MH798869.1 |
| *mphB* | CGCAGCGCTTGATCTTGTAG | TTACTGCATCCATACGCTGCTT | CGCAGCGCTTGATCTTGTAGTACACACACCAGAAGAAGCAAGAATGTCAATGAAGCAGCGTATGGATGCAGTAA | | 74 | "Mph(B) family macrolide 2'-phosphotransferase" : *Escherichia coli mph(B)* gene for Mph(B) family macrolide 2'-phosphotransferase : NG_047988.1 |
| *sat4* | GAATGGGCAAAGCATAAAAACTTG | CCGATTTTGAAACCACAATTATGATA | GAATGGGCAAAGCATAAAAACTTGCATGGACTAATGCTTGAAACCCAGGACAATAACCTTATAGCTTGTAAATTCTATCATAATTGTGGTTTCAAAATCGG | | 101 | "streptothricin N-acetyltransferase Sat4" : *Enterococcus faecalis* strain TY1 plasmid pDEF-1 : |
| *str* | AATGAGTTTTGGAGTGTCTCAACGTA | AATCAAAACCCCTATTAAAGCCAAT | AATGAGTTTTGGAGTGTCTCAACGTATGTAGCAAAGGGTGTTTTTAGAAGAGAAATATTATTTGCTTTAGATCATTTCAATAATATTTTACGTCCTGAATTATTAAGAATGATTTCTTGGTATATTGGCTTTAATAGGGGTTTTGATT | | 148 | "streptomycin adenylyltransferase Str" : *Staphylococcus aureus* strain CFSAN018749 plasmid pGMI14-005 : CP028191.1 |
| *strA* | CCGGTGGCATTTGAGAAAAA | GTGGCTCAACCTGCGAAAAG | GTGGCTCAACCTGCGAAAAGAAACGAGTTGCTATGGACTTGCACCGGTTGTGTTCCGGTCTATCTCTCATTTTAAGCGGCTTTTTTCTCAAATGCCACCGG | | 101 |  |
| *strB* | GCTCGGTCGTGAGAACAATCT | CAATTTCGGTCGCCTGGTAGT | CAATTTCGGTCGCCTGGTAGTCGCCGTGCTCGGCAACGATGTGAGAGAGCATTCGCTCCCCGGCATATTCGAGCAACATCAGATTGTTCTCACGACCGAGC | | 101 | "Streptomycin 3''-kinase (Streptomycin 3''-phosphotransferase) (SPH)" : *Acinetobacter baumannii* str. AYE : CU459141.1 (JohnsonmBio2016) |
| *tetX* | AAATTTGTTACCGACACGGAAGTT | CATAGCTGAAAAAATCCAGGACAGTT | CATAGCTGAAAAAATCCAGGACAGTTCACCTCTGGTTGATGAATATCGGCTTGTATATTGAAAGTACCTGTTTCTTCAACTTCCGTGTCGGTAACAAATTT | | 101 | "tetX - Kynurenine 3-monooxygenase" : *Escherichia coli* strain MS8345 plasmid pMS8345A : CP025402.1 |
| *ampC* | TGGCGTATCGGGTCAATGT | CTCCACGGGCCAGTTGAG | TGGCGTATCGGGTCAATGTATCAGGGTCTGGGCTGGGAGATGCTCAACTGGCCCGTGGAG | | 60bp | Beta-lactam (Cephalosporin) - class C betalactamase |
| *mphA* | CTGACGCGCTCCGTGTT | GGTGGTGCATGGCGATCT | CTGACGCGCTCCGTGTTGTCGATGAGCACATGGCCCACGTAGAGATCGCCATGCACCACC | | 60bp | macrolide - phosphotransferases |
| *ereB* | GCTTTATTTCAGGAGGCGGAAT | TTTTAAATGCCACAGCACAGAATC | GCTTTATTTCAGGAGGCGGAATGCAGGGCGATATGGGTGCAAAAGACAAATACATGGCAGATTCTGTGCTGTGGCATTTAAAA | | 83bp |  |
| *carB* | GGAGTGAGGCTGACCGTAGAAG | ATCGGCGAAACGCACAAA | GGAGTGAGGCTGACCGTAGAAGAAGAGTCCACGGGCAGAATTTCCTCGTCGACCGGGAAACAGTACAACGGTTTGTGCGTTTCGCCGAT | | 89 | "carbomycin resistance protein" : *Streptomyces thermotolerans* carbomycin resistance protein (*carB*) gene : M16503.1 |
| *ermB* | TAAAGGGCATTTAACGACGAAACT | TTTATACCTCTGTTTGTTAGGGAATTGAA | TTTATACCTCTGTTTGTTAGGGAATTGAAACTGTAGAATATCTTGGTGAATTAAAGTGACACGAGTATTCAGTTTTAATTTTTCTGACGATAAGTTGAATAGATGACTGTCTAATTCAATAGACGTTACCTGTTTACTTATTTTAGCCAGTTTCGTCGTTAAATGCCCTTTA | | 172 | "23S rRNA (adenine(2058)-N(6))-methyltransferase Erm(B)" : *Klebsiella* *pneumoniae* subsp. *pneumoniae* strain ARLG-3135 plasmid p2 : CP033948.1 |
| *ermF* | CAGCTTTGGTTGAACATTTACGAA | AAATTCCTAAAATCACAACCGACAA | CAGCTTTGGTTGAACATTTACGAAAATTATTTTCTGATGCCCGAAATGTTCAAGTTGTCGGTTGTGATTTTAGGAATTT | | 79 | "23S rRNA (adenine(2058)-N(6))-methyltransferase Erm(F)" : *Bacteroides thetaiotaomicron* : NG_047826.1 |
| *sul1* | CAGCGCTATGCGCTCAAG | ATCCCGTATGCGCTCAAG |  | |  |  |
| *sul2* | TCATCTGCCAAACTCGTCGTTA | GTCAAAGAACGCCGCAATGT | TCATCTGCCAAACTCGTCGTTATGCATTCGGTGCAAGACGGGCAGGCAGATCGGCGCGAGGCACCCGCTGGCGACATCATGGATCACATTGCGGCGTTCTTTGAC | | 105 | "sulfonamide-resistant dihydropteroate synthase Sul2" : *Salmonella* *enterica* subsp. *enterica* strain CFSA664 plasmid pCFSA664-1 : |
| *tet(M) (JUCL)* | TAATATTGGAGTTTTAGCTCATGTTGATG | CCTCTCTGACGTTCTAAAAGCGTATTAT |  | |  |  |
| *tet(O) (JUCL)* | ATGTGGATACTACAACGCATGAGATT | TGCCTCCACATGATATTTTTCCT | ATGTGGATACTACAACGCATGAGATTATACTTTCTTTTTTGGGGAATGTGCAGATGGAAGTCATTTGTGCCATCCTTGAGGAAAAATATCATGTGGAGGCA | | 101 | "tetO - tetracycline resistance protein" : *Campylobacter coli* strain ZTA14/01086 tetracycline resistance protein (tetO), phosphorylase (pnp), erythromycin resistance methylase B (*ermB*), and truncated TetO (*tetO*) genes : MF134831.1 |
| *tet(O)* | CAACATTAACGGAAAGTTTATTGTATACCA | TTGACGCTCCAAATTCATTGTATC | TTGACGCTCCAAATTCATTGTATCTGTCCTTGTTGTGCCTTCATCTACGCTCCCTAGTTCTGCAATTGCACCACTGGTATACAATAAACTTTCCGTTAATGTTG | | 104 | "Tetracycline resistance protein TetO" : *Salmonella* *enterica* strain 2016K-0796 plasmid p2016K-0796 : |
| *tet(Q)* | CGCCTCAGAAGTAAGTTCATACACTAAG | TCGTTCATGCGGATATTATCAGAAT | CGCCTCAGAAGTAAGTTCATACACTAAGGGCTTAGGCGTTTTTATGGTCAAGCCATGCGGGTATCAAATAACAAAAGGCGATTATTCTGATAATATCCGCATGAACGA | | 108 | "tetracycline resistance ribosomal protection protein Tet(Q)" : *Prevotella intermedia* PDRC-11 *tet(Q)* gene for tetracycline resistance ribosomal protection protein Tet(Q) : NG_048271.1 |
| *tet(W)* | ATGAACATTCCCACCGTTATCTTT | ATATCGGCGGAGAGCTTATCC | ATATCGGCGGAGAGCTTATCCCGAACAGACTGAACCACGCTCTGCAAATCAACGCCAGCCTGGTCGATCTTGTTGATAAAGATAACGGTGGGAATGTTCAT | | 101 | "Tetracycline resistance, ribosomal protection type" : *Streptococcus suis* strain BSB6 putative hydrolase (*hdy*) gene, complete cds; ICESsuBSB6 mobile element, complete sequence; and LSU ribosomal protein L7/L12 (*rplL*) gene : MF616023.1 |
| *tet(32)* | CCATTACTTCGGACAACGGTAGA | CAATCTCTGTGAGGGCATTTAACA | CCATTACTTCGGACAACGGTAGAGCCGCAAAAGCCGGAGCAAAGGGAAGCCCTGTTAAATGCCCTCACAGAGATTG | | 76 | "tetracycline resistance ribosomal protection protein Tet(32)" : *Clostridiaceae bacterium* K10 tet(32) gene for tetracycline resistance ribosomal protection protein Tet(32) : NG_048124.1 |
| *vanC1* | CCTGCCACAATCGATCGTT | CGGCTTCATTCGGCTTGATA | CCTGCCACAATCGATCGTTTTATTCAAGACCATGGATTCCCGATCTTTATCAAGCCGAATGAAGCCG | | 67 | "D-alanine--D-serine ligase VanC1" : *Enterococcus gallinarum* N04-0414 *vanC* gene : NG_048345 |
| *vanD* | CAGAGGAACATAATGTTTCGATAAAATCT | GCCGGATTTTGTGATTCCAA | CAGAGGAACATAATGTTTCGATAAAATCTGCGATGGAGATTGCCGCAAACATAGATACAAAAAAATATCAGCCTTATTATATTGGAATCACAAAATCCGGC | | 101 | "D-Ala:D-Lac ligase VanD" : *Enterococcus faecium* strain A902 vancomycin resistance gene cluster : EU999036.1 |
| *vanG* | ATTTGAATTGGCAGGTATACAGGTTA | ATTTGAATTGGCAGGTATACAGGTTA | ATTTGAATTGGCAGGTATACAGGTTATTGGATGTGGTACTCTTTCTTCAGCATTATGTATGGACAAAGACAAATCA | | 76 | "D-alanine--D-serine ligase VanG-Cd" : *Clostridioides difficile* 6616-NonSp/novelST *vanG* gene for D-alanine--D-serine ligase VanG-Cd : NG_061616.1 |
| *mcr-2* | CGACCAAGCCGAGTCTAAGG | CAACTGCGACCAACACACTT | CGACCAAGCCGAGTCTAAGGACTTGATGAATTTGGCGTTTTTTGTGCGAATTATCGGGCTTGGCGTGTTGCCAAGTGTGTTGGTCGCAGTTG | | 92bp | polymyxin E - phosphoethanolamine transferase family - target modification |
| *mcr-3* | ACCTCCAGCGTGAGATTGTTCCA | GCGGTTTCACCAACGACCAGAA | ACCTCCAGCGTGAGATTGTTCCAGCCAATTTCGTTAATAGTACCGTTAAATACGTTTACAATCGTTATCTTGCTGAACCAATCCCATTTACAACTTTAGGTGATGATGCAAAACGGGATACTAATCAAAGTAAGCCCACGTTGATGTTTCTGGTCGTTGGTGAAACCGC | | 169bp | polymyxin E - phosphoethanolamine transferase family - target modification |
| *ermC* | TTTGAAATCGGCTCAGGAAAA | ATGGTCTATTTCAATGGCAGTTACG | TTTGAAATCGGCTCAGGAAAAGGGCATTTTACCCTTGAATTAGTACAGAGGTGTAATTTCGTAACTGCCATTGAAATAGACCAT | | 84bp | MLS - Erm 23S rRNA methyltransferase |
| *czrC-2* | AATTGCCACAATCACAGCCG | CCCGATTGCAGCTAATCCCA | AATTGCCACAATCACAGCCGGCTACCATGTAATTATTCTCGAAGGAATTGGAGAGACAGTTGAAAATACTAAATTAAAGGGAAAATTCACTCCTAATTCTCATATTCTAATGGGATTAGCTGCAATCGGG | | 130 | "metal-transporting ATPase" : *Staphylococcus aureus* subsp. *aureus* strain LA-MRSA ST398 isolate E154 : MH188482 |
| *msrD (matA/mel)* | TAGTAGGCAAGCTCGGTGTTGA | CCTGTGCTATTTTAAGCCTTGTTTCT | TAGTAGGCAAGCTCGGTGTTGAGCAATTAAATATTCAGACCATGAGCGGTGGGGAAGAAACAAGGCTTAAAATAGCACAGG | | 81 | "msrD : ABC transporter involved in macrolide resistance" : *Streptococcus dysgalactiae* subsp. *equisimilis* comEC pseudogene, ORF1, ORF2, ORF3, *mef* gene, *msr(D)* gene and ORF6 (partial) : AM168138.1 |
| *mefA* | CCGTAGCATTGGAACAGCTTTT | AAACGGAGTATAAGAGTGCTGCAA | CCGTAGCATTGGAACAGCTTTTCACACCCCGGCTCTCAATGCGGTTACGCCACTTTTAGTACCAGAAGAACAGCTTACGAAATGTGCAGGCTATAGTCAGTCTTTGCAGTCTATAAGCTATATTGTTAGTCCGGCAGTTGCAGCACTCTTATACTCCGTTT | | 161 | "macrolide-efflux protein" : *Streptococcus suis* strain YY060816 composite mobile genetic element CMGEYY060816 mobile element : KX077898.1 |
| *tet(A)* | CTCACCAGCCTGACCTCGAT | CACGTTGTTATAGAAGCCGCATAG | CTCACCAGCCTGACCTCGATCGTCGGACCCCTCCTCTTCACGGCGATCTATGCGGCTTCTATAACAACGTG | | 70 | "tetracycline efflux MFS transporter Tet(A)" : *Klebsiella pneumoniae* subsp. *pneumoniae* strain CCRI-21711 plasmid pKp711-1 : CP035536 |
| *tet(B) (AFOL)* | AGTGCGCTTTGGATGCTGTA | AGCCCCAGTAGCTCCTGTGA | AGTGCGCTTTGGATGCTGTATTTAGGCCGTTTGCTTTCAGGGATCACAGGAGCTACTGGGGCT | | 63 | "tetB - Tetracycline efflux MFS transporter" : *Escherichia coli* strain DUK14-2 plasmid pMOO-32 : MK169211.1 |
| *tet(C) (JUCL)* | ACTGGTAAGGTA (SEKVENSEN MANGLER NOGET) | ATGCATAAACCA (SEKVENSEN MANGLER NOGET) |  | |  |  |
| *tet(L)* | ATGGTTGTAGTTGCGCGCTATAT | ATCGCTGGACCGACTCCTT | ATGGTTGTAGTTGCGCGCTATATTCCAAAGGAAAATAGGGGTAAAGCATTTGGTCTTATTGGATCGATAGTAGCCATGGGAGAAGGAGTCGGTCCAGCGAT | | 100 | "tetL - tetracycline efflux protein; confers tetracycline resistance" : *Staphylococcus chromogenes* strain Sch-734Lar plasmid pSch-734Lar : MH431899.1 |
| *tetPA* | AGTTGCAGATGTGTATAGTCGTAAACTATCTATT | TGCTACAAGTACGAAAACAAAACTAGAA | AGTTGCAGATGTGTATAGTCGTAAACTATCTATTGTTATTGGGGGAGTTTTAACAGGAGTGGGATTTATTTTAGAAGGTTCTATTTCTAGTTTTGTTTTCGTACTTGTAGCA | | 112 | "tetracycline efflux MFS transporter TetA(P)" : *Clostridium perfringens* CDH357 *tetA(P)* gene for tetracycline efflux MFS transporter TetA(P) : |
| *tolC1* | GGCCGAGAACCTGATGCA | AGACTTACGCAATTCCGGGTTA | GGCCGAGAACCTGATGCAAGTTTATCAGCAAGCACGCCTTAGTAACCCGGAATTGCGTAAGTCT | | 64 | "outer membrane channel protein TolC" : *Escherichia coli* strain Ec-2Lar chromosome : CP035318.1 |
| *qacEΔ1* | CCCCTTCCGCCGTTGT | CGACCAGACTGCATAAGCAACA | CCCCTTCCGCCGTTGTCATAATCGGTTATGGCATCGCATTTTATTTTCTTTCTCTGGTTCTGAAATCCATCCCTGTCGGTGTTGCTTATGCAGTCTGGTCG | | 101bp | disinfectant efflux pump |
| *mcr-1* | CGTTTATCATGCGTATCATTGG | AAGACTTGCCACGATCAAGC | CGTTTATCATGCGTATCATTGGTTTGGGTGTGCTACCAAGTTTGCTTGTGGCTTTTGTTAAGGTGGATTATCCGACTTGGGGCAAGGGTTTGATGCGCCGATTGGGCTTGATCGTGGCAAGTCTT | | 125 | "phosphoethanolamine--lipid A transferase MCR-1.1" : *Escherichia coli* strain CRE10 plasmid pCRE10.3 : CP034405.1 |
| *aac(6')-Ib 1* | GTTTGAGAGGCAAGGTACCGTAA | GAATGCCTGGCGTGTTTGA | GAATGCCTGGCGTGTTTGAACCATGTACACGGCTGGACCATCTGGGGTGGTTACGGTACCTTGCCTCTCAAAC | | 73 | "fluoroquinolone-acetylating aminoglycoside 6'-N-acetyltransferase AAC(6')-Ib-cr5": *K. pneumoniae* plasmid pKp711-3: CP035538 : (also gound inpCfSA 122-1) |
| *aac(6')-Ib 2* | AGAAGCACGCCCGACACTT | GCTCTCCATTCAGCATTGCA | GCTCTCCATTCAGCATTGCAATGTATGGAGTGACGGACTCTTGCGCTAAAACGCTTGGCAAGTACTGTTCCTGTACGTCAGCAAGTGTCGGGCGTGCTTCT | | 101 | "fluoroquinolone-acetylating aminoglycoside 6'-N-acetyltransferase AAC(6')-Ib-cr5" : *K. pneumoniae* plasmid pKp711-3 : CP035538 : (also found in pCfSA 122-1) |
| *aac(6')-II* | CGACCCGACTCCGAACAA | GCACGAATCCTGCCTTCTCA | GCACGAATCCTGCCTTCTCATAGCAGCGTATGGCTCGATGGTTGTTCGGAGTCGGGTCG | | 59 | "aminoglycoside 6'-N-acetyltransferase" : *Achromobacter xylosoxidans* : KT719387 |
| *aacA/aphD* | AGAGCCTTGGGAAGATGAAGTTT | TTGATCCATACCATAGACTATCTCATCA | TTGATCCATACCATAGACTATCTCATCAGTTTTTGGATAATGATAATCAGTATATAACTCATCATACATTTTATATATTTGTCCATATCCAATAGGAACATTGTTATATTCAATAATTACTCTAAAAACTTCATCTTCCCAAGGCTCT | | 148 | "bifunctional aminoglycoside modifying enzyme AacA-AphD" : gentamicin and kanamycin resistance e.g. *S. aureus* : MF185208 |
| *aacC1* | GGTCGTGAGTTCGGAGACGTA | GCAAGTTCCCGAGGTAATCG | GCAAGTTCCCGAGGTAATCGGAGTCCGGCTGATGTTGGGAGTAGGTGGCTACGTCTCCGAACTCACGACC | | 70 | "Gentamicin 3'-acetyltransferase (AAC(3)-I)" : plasmid RCS36_pI-II : LT985229 : E. coli |
| *aacC2* | ACGGCATTCTCGATTGCTTT | CCGAGCTTCACGTAAGCATTT | ACGGCATTCTCGATTGCTTTGCTATCGAAGGAAAGCCGGATGCGGTCGAAACTATAGCAAATGCTTACGTGAAGCTCGG | | 79 | "aminoglycoside 3-N-acetyltransferase AAC(3)-IIe" : *K. pneumoniae* plasmid pKp711-3 : CP035538 |
| *aadA1- B (aadA1 zhup)* | AGCTAAGCGCGAACTGCAAT | TGGCTCGAAGATACCTGCAA | TGGCTCGAAGATACCTGCAAGAATGTCATTGCGCTGCCATTCTCCAAATTGCAGTTCGCGCTTAGCT | | 67 | "ANT(3'')-Ia family aminoglycoside nucleotidyltransferase AadA1" : CP033224 : *Salmonella enterica* plasmid pCFSA 122-1 |
| *aadA5 1* | ATCACGATCTTGCGATTTTGCT | CTGCGGATGGGCCTAGAAG | ATCACGATCTTGCGATTTTGCTGACCAAGGCGAGGCAACACAGCCTTGCGCTTCTAGGCCCATCCGCAG | | 69 | "Streptomycin 3''-O-adenylyltransferase" : confers resistance to streptomycin and spectinomycin : MH990674.1 : salmonella : *E. coli* |
| *aadA5 2* | GTTCTTGCTCTTGCTCGCATT | GATGCTCGGCAGGCAAAC | GTTCTTGCTCTTGCTCGCATTTGGTACAGCGCTTCAACTGGTCTCATTGCTCCTAAGGACGTTGCTGCCGCATGGGTATCGGAGCGTTTGCCTGCCGAGCATC | | 103 | "Streptomycin 3''-O-adenylyltransferase" : confers resistance to streptomycin and spectinomycin : MH990674.1 : salmonella : *E. coli* |
| *aadA9 1* | CGCGGCAAGCCTATCTTG | CAAATCAGCGACCGCAGACT | CGCGGCAAGCCTATCTTGGGCTTTGCAAGGATAGTCTTGCTTTGCGTGCAGATGAGACTTCGGCGTTCATTGGCTATGCAAAGTCTGCGGTCGCTGATTTG | | 101 | "ANT(3'')-Ia family aminoglycoside nucleotidyltransferase AadA9" : *Corynebacterium glutamicum* LP-6 pTET3 *aadA9* gene : NG_047368.1 |
| *aadA9 2* | GGATGCACGCTTGGATGAA | CCTCTAGCGGCCGGAGTATT | GGATGCACGCTTGGATGAAGCTACCAGACGCTCCCTGATGCTCGATTTCTTGAATATCTCGGCACCACCATGCGAAAGCTCAATACTCCGGCCGCTAGAGG | | 101 | ANT(3'')-Ia family aminoglycoside nucleotidyltransferase AadA9 : *Corynebacterium glutamicum* LP-6 pTET3 *aadA9* gene : NG_047368.1 |
| *aph(2')-Id* | TAAGGATATACCGACAGTTTTGGAAA | TTTAATCCCTCTTCATACCAATCCATA | TAAGGATATACCGACAGTTTTGGAAAAATATATGATGAAAGAAAAATACTGGTCGTTCGAAAAGATTATCTATGGAAAGGAATATGGTTATATGGATTGGTATGAAGAGGGATTAAA | | 117 | "aminoglycoside resistance protein - APH(2'')-Id" : AY743255.1 : *Enterococcus faecium* APH(2'')-Id (*aph(2'')-Id*) gene |
| *aphA1* | TGAACAAGTCTGGAAAGAAATGCA | CCTATTAATTTCCCCTCGTCAAAAA | CCTATTAATTTCCCCTCGTCAAAAATAAGGTTATCAAGTGAGAAATCACCATGAGTGACGACTGAATCCGGTGAGAATGGCAAAAGCTTATGCATTTCTTTCCAGACTTGTTCA | | 114 | "aminoglycoside O-phosphotransferase APH(3')-Ia" : CP033224 : *Salmonella enterica* plasmid pCFSA 122-1 |
| *blaACC-1* | CACACAGCTGATGGCTTATCTAAAA | AATAAACGCGATGGGTTCCA | CACACAGCTGATGGCTTATCTAAAAGCATGGAAACCTGCCGATGCGGCTGGAACCCATCGCGTTTATT | | 68 | "cephalosporin-hydrolyzing class C beta-lactamase ACC-1" : *Salmonella* *enterica* subsp. *enterica* strain 15-SA01028 plasmid pSE15-SA01028 : CP026661.1 |
| *blaCMY-2* | CCGCGGCGAAATTAAGC | GCCACTGTTTGCCTGTCAGTT | CCGCGGCGAAATTAAGCTCAGCGATCCGGTCACGAAATACTGGCCAGAACTGACAGGCAAACAGTGGC | | 68 | "class C beta-lactamase CMY-2" : *Salmonella* *enterica* subsp. *enterica* serovar Newport str. CDC 2012K-0663 plasmid pSNE2-2012K-0663 : CP025245.1 |
| *CMYX (blaCMY2a)* | GCGAGCAGCCTGAAGCA | CGGATGGGCTTGTCCTCTT | GCGAGCAGCCTGAAGCAGCCATTTGCCCAGTTGATGGAGCAGACCCTGCTGCCCGGGCTCGGCATGCACCACACCTATGTCAATGTGCCGAAGCAGGCCATGGCGAGTTATGCCTATGGCTATTCGAAAGAGGACAAGCCCATCCG | | 146 | "CMY-1/MOX family class C extended-spectrum beta-lactamase CMY-9" : *Escherichia coli* HKYM68 pCMXR1 blaMOX gene : NG_048888.1 (blast: blaCMY9/8/10/11 osv.) |
| *blaCTX-Ma* | GGAGGCGTGACGGCTTTT | TTCAGTGCGATCCAGACGAA | GGAGGCGTGACGGCTTTTGCCCGCGCGATCGGCGATGAGACGTTTCGTCTGGATCGCACTGAA | | 63 | "class A extended-spectrum beta-lactamase CTX-M-215" : *Escherichia coli* EC039 *blaCTX-M* gene : NG_063838.1 |
| *blaCTX-Mb* | GCCGCGGTGCTGAAGA | ATCGGATTATAGTTAACCAGGTCAGATTT | GCCGCGGTGCTGAAGAAAAGTGAAAGCGAACCGAGTCTGTTAAATCAGCGAGTTGAGATCAAAAAATCTGACCTGGTTAACTATAATCCGAT | | 92 | "class A extended-spectrum beta-lactamase CTX-M-60" : *Klebsiella pneumoniae* *blaCTX-M* gene for class A extended-spectrum beta-lactamase CTX-M-60 : NG_049011.1 |
| *blaCTX-Mc* | CGATACCACCACGCCGTTA | GCATTGCCCAACGTCAGATT | CGATACCACCACGCCGTTAGCGATGGCGCAGGCTCTGCGCAATCTGACGTTGGGCAATGC | | 60 | "class A extended-spectrum beta-lactamase CTX-M-217" : *Providencia stuartii* 1700431 *blaCTX-M* gene for class A extended-spectrum beta-lactamase CTX-M-217 : NG_057610.1 |
| *blaCTX-Md* | CTTGGCGTTGCGCTGAT | CGTTCATCGGCACGGTAGA | CTTGGCGTTGCGCTGATTAACACCGCCGATAATTCGCAGATTCTCTACCGTGCCGATGAACG | | 62 | "extended spectrum beta-lactamase CTX-M" : *Escherichia coli* strain 1481 extended spectrum beta-lactamase *CTX-M* gene : MG581457.1 |
| *blaCTX-Me* | GCGATAACGTGGCGATGAAT | GTCGAGACGGAACGTTTCGT | GCGATAACGTGGCGATGAATAAGCTGATTGCTCACGTTGGCGGCCCGGCTAGCGTCACCGCGTTCGCCCGACAGCTGGGAGACGAAACGTTCCGTCTCGAC | | 101 | "class A extended-spectrum beta-lactamase CTX-M-60" : Klebsiella pneumoniae *blaCTX-M* gene for class A extended-spectrum beta-lactamase CTX-M-60 : NG_049011.1 (findes også på plasmid pNH34.1 - CP034406.1 ) |
| *bla-L1* | CACCGGGTTACCAGCTGAAG | GCGAAGCTGCGCTTGTAGTC | CACCGGGTTACCAGCTGAAGGGCAATCCCCGTTATCCGCGCCTGATCGAGGACTACAAGCGCAGCTTCGC | | 70 | "L1 family subclass B3 metallo-beta-lactamase" : *Stenotrophomonas maltophilia* *blaL1* gene for L1 family subclass B3 metallo-beta-lactamase : NG_047517.1 |
| *blaOXA1/blaOXA30* | CGGATGGTTTGAAGGGTTTATTAT | TCTTGGCTTTTATGCTTGATGTTAA | TCTTGGCTTTTATGCTTGATGTTAAATTCGACCCCAAGTTTCCTGTAAGTGCGGACACAAAAACATATTTATGTCCTGATTTGCTTATAATAAACCCTTCAAACCATCCG | | 110 | "oxacillin-hydrolyzing class D beta-lactamase OXA-1" : *Klebsiella pneumoniae* strain NH34 plasmid pNH34.1 : CP034406.1 |
| *blaOXA10 1* | CGCAATTATCGGCCTAGAAACT | TTGGCTTTCCGTCCCATTT | TTGGCTTTCCGTCCCATTTGAAAACCTGATGCTCATTCTTTATGACACCAGTTTCTAGGCCGATAATTGCG | | 70 | "oxacillin-hydrolyzing class D beta-lactamase OXA-10" : *Escherichia coli* strain SCEC020023 plasmid pOXA10_020023 : CP025944 |
| *blaPER* | TGCTGGTTGCTGTTTTTGTGA | CCTGCGCAATGATAGCTTCAT | CCTGCGCAATGATAGCTTCATTGGTTCGGCTTGACTCGGCTGAGTCTTTCACAAAAACAGCAACCAGCA | | 69 | "extended-spectrum beta-lactamase PER-1" : *Pseudomonas aeruginosa* *PER-1* gene for extended-spectrum beta-lactamase PER-1 : Z21957.1 |
| *blaVIM* | GCACTTCTCGCGGAGATTG | CGACGGTGATGCGTACGTT | GCACTTCTCGCGGAGATTGAGAAGCAAATTGGACTTCCTGTAACGCGTGCAGTCTCCACGCACTTTCATGACGACCGCGTCGGCGGCGTTGATGTCCTTCGGGCGGCTGGGGTGGCAACGTACGCATCACCGTCG | | 135 | "subclass B1 metallo-beta-lactamase VIM-62" : *Pseudomonas putida* 174364 *blaVIM* gene for subclass B1 metallo-beta-lactamase VIM-62 : NG_063896.1 |
| *blaSHV* | GCTGGAGCGAAAGATCCACT | CGCCTCATTCAGTTCCGTTT | CGCCTCATTCAGTTCCGTTTCCCAGCGGTCAAGGCGGGTGACGTTGTCGCCGATCTGGCGCAAAAAGGCAGTCAATCCTGCGGGGCCGCCGACGGTGGCCAGCAGCAGATTGGCGGCGCTGTTATCGCTCATGGTAATGGCGGCGGCGCAGAGTTCGCCGACCGTCATGCCGTCGGCAAGGTGTTTTTCGCTGACCGGCGAGTAGTCCACCAGATCCTGCTGGCGATAGTGGATCTTTCGCTCCAGC | | 247 | "Bla beta-lactamase SHV-2" : *Escherichia coli* strain MB6212 plasmid pMB5876 : MK070495.1 |
| *cphA(1)* | GCGAGCTGCACAAGCTGAT | CGGCCCAGTCGCTCTTC | GCGAGCTGCACAAGCTGATCAAACGGGTCAGCCGCCAGCCGGTGCTGGAGGTGATCAACACCAACTACCACACCGACCGGGCGGGCGGTAACGCCTACTGGAAGTCCATCGGGGCCAAGGTGGTCTCGACCCGCCAGACCCGGGATCTGATGAAGAGCGACTGGGCCG | | 168 | "CphA-type class B metallo-beta-lactamase" : *Aeromonas hydrophila* *cphA* gene for CphA-type class B metallo-beta-lactamase : LC270625.1 |
| *czrC-1* | CACAGCCGGCTACCATGTAA | ATCAGAGAAGCCCCGATTGC | CACAGCCGGCTACCATGTAATTATTCTCGAAGGAATTGGAGAGACAGTTGAAAATACTAAATTAAAGGGAAAATTCACTCCTAATTCTCATATTCTAATGGGATTAGCTGCAATCGGGGCTTCTCTGAT | | 129 | "metal-transporting ATPase" : *Staphylococcus aureus* subsp. *aureus* strain LA-MRSA ST398 isolate E154 : MH188482 |
| *lnuC* | TGGTCAATATAACAGATGTAAACCAGATTT | CACCCCAGCCACCATCAA | TGGTCAATATAACAGATGTAAACCAGATTTTCCAATTTGCAATAGATGCGGAGATTAAAGTCTTTCTTGATGGTGGCTGGGGTG | | 84 | "lincosamide nucleotidyltransferase Lnu(C)" : *Streptococcus agalactiae* UCN36 *lnu(C)* gene for lincosamide nucleotidyltransferase Lnu(C) : NG_047924.1 |
| *IS6100* | CCGATCACGGAAAGCTCAAG | GGCTCGCATGACTTCGAATC | | GGCTCGCATGACTTCGAATCCCTTGATCGTGGCATAGGCCGTGGGGATCGATTTGAAACCGCGCACCGGCTTGATCAGTATCTTGAGCTTTCCGTGATCGG | 101 |  |
| *intI1* | TGCCGTGATCGAAATCCAGATCCT | TTTCTGGAAGGCGAGCATCGTTTG | TGCCGTGATCGAAATCCAGATCCTTGACCCGCAGTTGCAAACCCTCACTGATCCGCATGCCCGTTCCATACAGAAGCTGGGCGAACAAACGATGCTCGCCTTCCAGAAA | | 109bp | Class 1 integrons |
| *tp614* | GGAAATCAACGGCATCCAGTT | CATCCATGCGCTTTTGTCTCT | GGAAATCAACGGCATCCAGTTTGAACTCAATTCCATTCTTGTTGAGAAATGGGAAGGCAAGTGCTATCGTCTTGTCATTCAGAGACAAAAGCGCATGGATG | | 101bp |  |
| *is4* | GGGCGGGTCGATTGAAA | GTGGGCGGGATCTGCTT | GGGCGGGTCGATTGAAAACGGCGTTCTGCATATCGAGAAACTCGAAGCCGCCGCGCCGAC AGGCGCCGAAGATCTGGTGCTCGATCTCTACAAGCAGATCCCGCCCAC | | 108bp |  |
| *IS26* | GCCGCACTGTCGATTTTTATC | GCGGGATCTGCCACTTCTT | | GCGGGATCTGCCACTTCTTCACGTTGTTGAGGATTTTACCCAGAAACCGGTATGCAGCTTTGCTGTTACGACGGGAGGAGAGATAAAAATCGACAGTGCGGC | 102 |  |
| *IncNrepA164* | AGTTCACCACCTACTCGCTCCG | CAAGTTCTTCTGTTGGGATTCCG | AGTTCACCACCTACTCGCTCCGCGATTGTGGCAGTCTTCGAAATCCACGGACGATCCGCCTTTATGAAAGTCTTGCTCAATTCAAATCTTCAGGCTTATGGGTTACTACTCATGCTTGGTTAAATGACCGTTTCCTTTTGCCGGAATCCCAACAGAAGAACTTG | | 164 | "RepB family plasmid replication initiator protein" : *Salmonella enterica* subsp. *enterica* serovar Newport str. USDA-ARS-USMARC-1925 plasmid pSNE1-1925 : CP025233 |
| *IS1216* | TGCAGATGGTTTAACCTTGGATATTT | TCGGTTCATCAAACTGCTTCAC | | TCGGTTCATCAAACTGCTTCACTAACCGCTTAAGAAAAGCATAGGCTGCTTGTGTGTCCCGTTTTTTACGTAACCAAATATCCAAGGTTAAACCATCTGCA | 101 |  |

**Table S3:** ASVs significantly different between treated and untreated pigs for at least two consecutive samplings. Beta is the regression coefficient of ANCOM-BC.

| **Week** | **ASV** | **beta_values** | **Highest abundance** | **p-value** |
| --- | --- | --- | --- | --- |
| Week 02 | Bacteria_Firmicutes_Bacilli_Erysipelotrichales_Erysipelotrichaceae_[Clostridium] innocuum group_NA | -2.423338312 | Treated | 1.72675E-14 |
| Week 04 | Bacteria_Firmicutes_Bacilli_Erysipelotrichales_Erysipelotrichaceae_[Clostridium] innocuum group_NA | -1.323827284 | Treated | 1.11041E-05 |
| Week 02 | Bacteria_Firmicutes_Bacilli_Lactobacillales_Enterococcaceae_Enterococcus_casseliflavus/faecium/gallinarum/saccharolyticus | -3.830255305 | Treated | 1.84412E-08 |
| Week 04 | Bacteria_Firmicutes_Bacilli_Lactobacillales_Enterococcaceae_Enterococcus_casseliflavus/faecium/gallinarum/saccharolyticus | -3.136305228 | Treated | 4.72892E-08 |
| Week 02 | Bacteria_Firmicutes_Bacilli_Lactobacillales_Enterococcaceae_Enterococcus_NA.1 | -2.720938845 | Treated | 3.94878E-08 |
| Week 04 | Bacteria_Firmicutes_Bacilli_Lactobacillales_Enterococcaceae_Enterococcus_NA.1 | -1.618647932 | Treated | 0.000111972 |
| Week 02 | Bacteria_Firmicutes_Clostridia_Lachnospirales_Lachnospiraceae_[Eubacterium] fissicatena group_NA.3 | -1.451787867 | Treated | 3.70853E-06 |
| Week 04 | Bacteria_Firmicutes_Clostridia_Lachnospirales_Lachnospiraceae_[Eubacterium] fissicatena group_NA.3 | -1.336678856 | Treated | 1.57055E-05 |
| Week 05 | Bacteria_Firmicutes_Clostridia_Lachnospirales_Lachnospiraceae_[Ruminococcus] gauvreauii group_NA.2 | -1.684202049 | Treated | 5.17546E-06 |
| Week 07 | Bacteria_Firmicutes_Clostridia_Lachnospirales_Lachnospiraceae_[Ruminococcus] gauvreauii group_NA.2 | -0.940750009 | Treated | 5.76362E-05 |
| Week 02 | Bacteria_Firmicutes_Clostridia_Lachnospirales_Lachnospiraceae_Hungatella_hathewayi | -3.079015419 | Treated | 5.65416E-14 |
| Week 04 | Bacteria_Firmicutes_Clostridia_Lachnospirales_Lachnospiraceae_Hungatella_hathewayi | -2.626810725 | Treated | 7.85489E-09 |
| Week 02 | Bacteria_Firmicutes_Clostridia_Lachnospirales_Lachnospiraceae_Hungatella_NA | -2.361607393 | Treated | 5.6147E-10 |
| Week 04 | Bacteria_Firmicutes_Clostridia_Lachnospirales_Lachnospiraceae_Hungatella_NA | -2.108450274 | Treated | 1.23535E-06 |
| Week 06 | Bacteria_Firmicutes_Clostridia_Oscillospirales_Oscillospiraceae_Colidextribacter_NA | -1.489168866 | Treated | 1.64157E-06 |
| Week 07 | Bacteria_Firmicutes_Clostridia_Oscillospirales_Oscillospiraceae_Colidextribacter_NA | -1.101823489 | Treated | 1.15424E-06 |
| Week 04 | Bacteria_Firmicutes_Clostridia_Peptostreptococcales-Tissierellales_Anaerovoracaceae_[Eubacterium] nodatum group_NA | -1.461777762 | Treated | 5.63811E-05 |
| Week 05 | Bacteria_Firmicutes_Clostridia_Peptostreptococcales-Tissierellales_Anaerovoracaceae_[Eubacterium] nodatum group_NA | -1.120058602 | Treated | 1.67515E-05 |
| Week 07 | Bacteria_Bacteroidota_Bacteroidia_Bacteroidales_Tannerellaceae_Parabacteroides_NA | 1.62726621 | Untreated | 2.17046E-06 |
| Week 08 | Bacteria_Bacteroidota_Bacteroidia_Bacteroidales_Tannerellaceae_Parabacteroides_NA | 1.474108167 | Untreated | 3.48977E-06 |
| Week 04 | Bacteria_Firmicutes_Bacilli_Lactobacillales_Streptococcaceae_Streptococcus_suis | 1.676437869 | Untreated | 0.000116478 |
| Week 05 | Bacteria_Firmicutes_Bacilli_Lactobacillales_Streptococcaceae_Streptococcus_suis | 2.147758408 | Untreated | 2.61158E-06 |
| Week 02 | Bacteria_Firmicutes_Clostridia_Christensenellales_Christensenellaceae_Christensenellaceae R-7 group_NA.13 | 1.591436945 | Untreated | 1.62812E-08 |
| Week 04 | Bacteria_Firmicutes_Clostridia_Christensenellales_Christensenellaceae_Christensenellaceae R-7 group_NA.13 | 1.618561667 | Untreated | 5.39579E-07 |
| Week 02 | Bacteria_Fusobacteriota_Fusobacteriia_Fusobacteriales_Fusobacteriaceae_Fusobacterium_gastrosuis | 1.80122001 | Untreated | 2.41974E-06 |
| Week 04 | Bacteria_Fusobacteriota_Fusobacteriia_Fusobacteriales_Fusobacteriaceae_Fusobacterium_gastrosuis | 1.602561967 | Untreated | 1.88782E-09 |
| Week 05 | Bacteria_Fusobacteriota_Fusobacteriia_Fusobacteriales_Fusobacteriaceae_Fusobacterium_gastrosuis | 1.1614771 | Untreated | 7.29732E-05 |
| Week 05 | Bacteria_Verrucomicrobiota_Chlamydiae_Chlamydiales_Chlamydiaceae_Chlamydia_suis | 2.327278996 | Untreated | 3.30131E-07 |
| Week 06 | Bacteria_Verrucomicrobiota_Chlamydiae_Chlamydiales_Chlamydiaceae_Chlamydia_suis | 1.516469441 | Untreated | 3.43614E-05 |

**Table S4:** Genera significantly different between treated and untreated pigs for at least two consecutive samplings. Beta is the regression coefficient of ANCOM-BC.

| **Week** | **Genus** | **beta_values** | **Highest abundance** | **p-value** |
| --- | --- | --- | --- | --- |
| Week 02 | Bacteria_Firmicutes_Bacilli_Erysipelotrichales_Erysipelotrichaceae_[Clostridium] innocuum group | -2.28713 | Treated | 5.66E-12 |
| Week 04 | Bacteria_Firmicutes_Bacilli_Erysipelotrichales_Erysipelotrichaceae_[Clostridium] innocuum group | -1.44036 | Treated | 4.23E-06 |
| Week 02 | Bacteria_Firmicutes_Bacilli_Lactobacillales_Enterococcaceae_Enterococcus | -3.07665 | Treated | 2.45E-07 |
| Week 04 | Bacteria_Firmicutes_Bacilli_Lactobacillales_Enterococcaceae_Enterococcus | -3.19279 | Treated | 1.55E-08 |
| Week 02 | Bacteria_Firmicutes_Bacilli_Staphylococcales_Staphylococcaceae_Staphylococcus | -1.54352 | Treated | 0.00011 |
| Week 04 | Bacteria_Firmicutes_Bacilli_Staphylococcales_Staphylococcaceae_Staphylococcus | -1.89759 | Treated | 1.91E-06 |
| Week 02 | Bacteria_Firmicutes_Clostridia_Lachnospirales_Lachnospiraceae_[Eubacterium] fissicatena group | -1.78992 | Treated | 2.37E-05 |
| Week 04 | Bacteria_Firmicutes_Clostridia_Lachnospirales_Lachnospiraceae_[Eubacterium] fissicatena group | -2.00311 | Treated | 0.00011 |
| Week 04 | Bacteria_Firmicutes_Clostridia_Lachnospirales_Lachnospiraceae_Eisenbergiella | -2.46362 | Treated | 1.53E-08 |
| Week 05 | Bacteria_Firmicutes_Clostridia_Lachnospirales_Lachnospiraceae_Eisenbergiella | -1.16074 | Treated | 0.000122 |
| Week 02 | Bacteria_Firmicutes_Clostridia_Lachnospirales_Lachnospiraceae_Hungatella | -3.3817 | Treated | 1.62E-15 |
| Week 04 | Bacteria_Firmicutes_Clostridia_Lachnospirales_Lachnospiraceae_Hungatella | -2.94316 | Treated | 5.47E-09 |
| Week 05 | Bacteria_Firmicutes_Clostridia_Oscillospirales_Hydrogenoanaerobacterium_NA | -0.91801 | Treated | 6E-05 |
| Week 06 | Bacteria_Firmicutes_Clostridia_Oscillospirales_Oscillospiraceae_Intestinimonas | -1.0663 | Treated | 0.000176 |
| Week 07 | Bacteria_Firmicutes_Clostridia_Oscillospirales_Oscillospiraceae_Intestinimonas | -0.87271 | Treated | 7.93E-05 |
| Week 04 | Bacteria_Firmicutes_Clostridia_Oscillospirales_Ruminococcaceae_UBA1819 | -1.77612 | Treated | 2.21E-06 |
| Week 06 | Bacteria_Firmicutes_Clostridia_Oscillospirales_Ruminococcaceae_UBA1819 | -0.6172 | Treated | 0.000253 |
| Week 05 | Bacteria_Firmicutes_Clostridia_Peptococcales_Peptococcaceae_NA | -1.05534 | Treated | 0.000102 |
| Week 04 | Bacteria_Firmicutes_Clostridia_Peptostreptococcales-Tissierellales_Anaerovoracaceae_[Eubacterium] nodatum group | -1.45104 | Treated | 8.67E-05 |
| Week 05 | Bacteria_Firmicutes_Clostridia_Peptostreptococcales-Tissierellales_Anaerovoracaceae_[Eubacterium] nodatum group | -1.18452 | Treated | 4.64E-05 |
| Week 06 | Bacteria_Firmicutes_Clostridia_Peptostreptococcales-Tissierellales_Anaerovoracaceae_Family XIII AD3011 group | -0.62875 | Treated | 6.92E-06 |
| Week 05 | Bacteria_Proteobacteria_Gammaproteobacteria_Burkholderiales_Oxalobacteraceae_Oxalobacter | -0.46285 | Treated | 0.00015 |
| Week 02 | Bacteria_Bacteroidota_Bacteroidia_Bacteroidales_Prevotellaceae_Prevotella | 2.02628 | Untreated | 2.62E-07 |
| Week 04 | Bacteria_Bacteroidota_Bacteroidia_Bacteroidales_Prevotellaceae_Prevotella | 1.781627 | Untreated | 7.97E-05 |
| Week 07 | Bacteria_Bacteroidota_Bacteroidia_Bacteroidales_Prevotellaceae_Prevotella | 0.576638 | Untreated | 0.000221 |
| Week 07 | Bacteria_Bacteroidota_Bacteroidia_Bacteroidales_Rikenellaceae_Rikenellaceae RC9 gut group | 1.568962 | Untreated | 5.7E-15 |
| Week 08 | Bacteria_Bacteroidota_Bacteroidia_Bacteroidales_Rikenellaceae_Rikenellaceae RC9 gut group | 0.711574 | Untreated | 7.78E-05 |
| Week 02 | Bacteria_Desulfobacterota_Desulfovibrionia_Desulfovibrionales_Desulfovibrionaceae_Desulfovibrio | 2.925096 | Untreated | 6.56E-29 |
| Week 04 | Bacteria_Desulfobacterota_Desulfovibrionia_Desulfovibrionales_Desulfovibrionaceae_Desulfovibrio | 1.576412 | Untreated | 2.67E-05 |
| Week 07 | Bacteria_Desulfobacterota_Desulfovibrionia_Desulfovibrionales_Desulfovibrionaceae_Desulfovibrio | 1.180832 | Untreated | 1.59E-05 |
| Week 07 | Bacteria_Firmicutes_Bacilli_Erysipelotrichales_Erysipelatoclostridiaceae_Erysipelotrichaceae UCG-003 | 0.721923 | Untreated | 1.37E-07 |
| Week 14 | Bacteria_Firmicutes_Bacilli_Erysipelotrichales_Erysipelatoclostridiaceae_Erysipelotrichaceae UCG-003 | 0.648098 | Untreated | 4.75E-05 |
| Week 02 | Bacteria_Firmicutes_Clostridia_Christensenellales_Christensenellaceae_Christensenellaceae R-7 group | 2.311795 | Untreated | 2.2E-10 |
| Week 04 | Bacteria_Firmicutes_Clostridia_Christensenellales_Christensenellaceae_Christensenellaceae R-7 group | 2.333059 | Untreated | 4.11E-07 |
| Week 07 | Bacteria_Firmicutes_Clostridia_Lachnospirales_Lachnospiraceae_Eisenbergiella | 0.841669 | Untreated | 9.3E-08 |
| Week 02 | Bacteria_Firmicutes_Clostridia_Oscillospirales_Hydrogenoanaerobacterium_NA | 0.881979 | Untreated | 0.000103 |
| Week 02 | Bacteria_Firmicutes_Clostridia_Oscillospirales_Oscillospiraceae_Intestinimonas | 1.079934 | Untreated | 0.000425 |
| Week 04 | Bacteria_Firmicutes_Clostridia_Oscillospirales_Ruminococcaceae_Candidatus Soleaferrea | 1.132548 | Untreated | 0.00028 |
| Week 07 | Bacteria_Firmicutes_Clostridia_Oscillospirales_Ruminococcaceae_Candidatus Soleaferrea | 0.836039 | Untreated | 0.000467 |
| Week 07 | Bacteria_Firmicutes_Clostridia_Peptococcales_Peptococcaceae_NA | 0.71587 | Untreated | 0.000297 |
| Week 05 | Bacteria_Firmicutes_Clostridia_Peptostreptococcales-Tissierellales_Anaerococcus_NA | 1.250402 | Untreated | 0.000131 |
| Week 07 | Bacteria_Firmicutes_Clostridia_Peptostreptococcales-Tissierellales_Anaerococcus_NA | 0.767963 | Untreated | 2.75E-05 |
| Week 02 | Bacteria_Firmicutes_Clostridia_Peptostreptococcales-Tissierellales_Anaerovoracaceae_Family XIII AD3011 group | 1.147323 | Untreated | 3.64E-06 |
| Week 02 | Bacteria_Fusobacteriota_Fusobacteriia_Fusobacteriales_Fusobacteriaceae_Fusobacterium | 1.99071 | Untreated | 9.76E-07 |
| Week 04 | Bacteria_Fusobacteriota_Fusobacteriia_Fusobacteriales_Fusobacteriaceae_Fusobacterium | 1.741158 | Untreated | 7E-09 |
| Week 07 | Bacteria_Proteobacteria_Gammaproteobacteria_Burkholderiales_Oxalobacteraceae_Oxalobacter | 0.673231 | Untreated | 8.56E-07 |
| Week 05 | Bacteria_Verrucomicrobiota_Chlamydiae_Chlamydiales_Chlamydiaceae_Chlamydia | 2.349692 | Untreated | 2.69E-07 |
| Week 06 | Bacteria_Verrucomicrobiota_Chlamydiae_Chlamydiales_Chlamydiaceae_Chlamydia | 1.481629 | Untreated | 5.3E-05 |
